# Planthopper bugs use a fast, cyclic elastic recoil mechanism for effective vibrational communication at small body size

**DOI:** 10.1371/journal.pbio.3000155

**Published:** 2019-03-12

**Authors:** Leonidas-Romanos Davranoglou, Alice Cicirello, Graham K. Taylor, Beth Mortimer

**Affiliations:** 1 Department of Zoology, University of Oxford, Oxford, United Kingdom; 2 Department of Engineering Science, University of Oxford, Oxford, United Kingdom; 3 School of Biological Sciences, University of Bristol, Bristol, United Kingdom; University of Maryland, UNITED STATES

## Abstract

Vibrations through substrates are an important source of information for diverse organisms, from nematodes to elephants. The fundamental challenge for small animals using vibrational communication is to move their limited mass fast enough to provide sufficient kinetic energy for effective information transfer through the substrate whilst optimising energy efficiency over repeated cycles. Here, we describe a vibratory organ found across a commercially important group of plant-feeding insects, the planthoppers (Hemiptera: Fulgoromorpha). This elastic recoil snapping organ generates substrate-borne broadband vibrations using fast, cyclical abdominal motion that transfers kinetic energy to the substrate through the legs. Elastic potential energy is stored and released twice using two different latched energy-storage mechanisms, each utilising a different form of elastic recoil to increase the speed of motion. Comparison to the acoustic tymbal organ of cicadas (Hemiptera: Cicadomorpha) reveals functional convergence in their use of elastic mechanisms to increase the efficacy of mechanical communication.

## Abstract 2

Planthopper insects produce fast abdominal twerks for vibrational communication through the substrate, employing a novel vibratory organ that uses two reciprocal elastic recoil mechanisms to generate fast cyclical motion.

## Author summary

Animals use substrate-borne vibrations for eavesdropping and communication over an immense range of body size—from elephants to nematodes. Vibrational communication is especially challenging for small animals because of the high mechanical power that is needed to transmit information effectively over extended distances through a substrate. Here, we show that planthoppers, a commercially important group of insects, produce vibrations for communication using a reciprocal elastic recoil mechanism that proves remarkably effective at small body size. By combining morphological and biomechanical analyses of a previously overlooked vibratory organ on the abdomen, we show that planthoppers use fast, cyclical abdominal motions to generate substrate-borne vibrations. This novel, to our knowledge, mechanism, which we term the snapping organ, makes use of slow energy storage and fast elastic recoil twice during each cycle of motion, involving two distinct elastic elements. This cyclical mechanism allows planthoppers to transmit signal pulses containing a broad range of frequencies to the substrate. The mechanism is efficient, achieving fast cyclical motion without relying on high muscle power and mass, both of which are limited for animals of small size. The snapping organ is ubiquitous across planthoppers and presents an interesting example of how elastic mechanisms can be used to enable nonacoustic vibrational communication between animals.

Information transfer involving substrate-borne vibrations along surfaces or through materials is important to a wide variety of taxa, from elephants to nematode worms [1]. The key challenge for successful vibration generation lies in balancing energy-efficient motion for repeated signalling [2] with effective and robust information transfer [3]. Signalling efficiency can be optimised by minimising the frequency of active muscle contraction [4], whereas signalling efficacy is optimised by maximising the kinetic energy transferred to the substrate. One solution to this tradeoff, as we show here, is to make use of elastic recoil mechanisms in which elastic energy is stored slowly and then quickly released. This is sometimes referred to informally as power amplification because the time over which work is performed is reduced [2], although this is not true amplification in the sense of adding energy into the system from an outside source. This rapid release of energy is essential because kinetic energy scales with mass and with speed squared such that signalling efficacy is increased by producing faster, higher-amplitude motions that improve the chances of the signal reaching and stimulating its potential receivers. A further reason for favouring faster motions is that broadband signals are more robust to frequency-based filtering and environmental noise than are narrowband signals [5]: for mechanical impulses, or taps, a higher speed of motion increases the frequency content of the signal by producing a sharper impulse [6]. Frequency filtering and noise level will vary with the physical properties of the substrate [5].

Achieving the fast motions needed for effective vibrational communication is a particular challenge for smaller animals. Other things being equal, their lower mass means that faster speeds are needed to transfer kinetic energy to the substrate. Smaller animals also have shorter lever arms that limit output speed and amplitude for a given motor input, and their smaller muscles have limited potential for high motor input through direct muscle action [7]. Natural mechanisms for increasing the speed of motion, especially in smaller animals, involve elastic recoil mechanisms in which energy is elastically stored slowly and released quickly. This is particularly well studied for one-off ballistic motions such as the closing of ant jaws [8] or mantis shrimp claws [9], the projecting of toad tongues [10], or the jumping of froghoppers [11]. Much less is known about whether and how biological systems use elastic recoil to achieve very fast cyclical motions, in which the added challenge is to accommodate this within an efficient cycle of multidirectional motion. Perhaps the only good example of elastic recoil cyclical motion is the buckling of the drum-like tymbal organ of cicadas, which can generate loud acoustic vibration through an efficient bistable motion [12]. Insect flight provides another example of elastic energy storage in a fast oscillatory system, but the cyclical motions of the flight motor are mainly optimised for smooth transfer of kinetic and potential energy through the cycle, producing a nearly sinusoidal motion of the wingtips in a typical insect such as a hoverfly [13]. In contrast, vibration generation in the cicada’s buckling tymbal organ relies on the sudden release of energy [2]. Good examples from other contexts are lacking, meaning that general insights into how biological systems overcome these challenges have yet to be drawn. This leads to the fundamental research question that we set out to answer in this study: how do very small animals achieve the very fast motions needed for effective and efficient vibration generation?

Hemiptera, or true bugs, have expanded the use of vibrational signalling more than any other insect order [14]. Although there is a large and growing body of research into the behavioural ecology of vibrational communication [1], there are few studies detailing the mechanisms by which these enigmatic vibrations are generated. Hemiptera are known to generate vibrations in various ways, ranging from the use of buckling tymbals (ribs that pop between bent and straight conformations) [15] or stridulatory structures (body parts that are rubbed together, often as a scraper and a file) [16] to the use of wing buzzing [17], leg drumming [18], and tremulation (vibration of the body relative to legs) [17, 19]. With the exception of tremulation, which does not generate much acoustic vibration, these various mechanisms all emit acoustic and substrate-bound vibrations simultaneously [20]. Here, we report a novel, to our knowledge, vibratory organ, the snapping organ, in planthoppers (Hemiptera: Fulgoromorpha). These bugs are a speciose infraorder comprising over 12,500 described species [21] and containing several economically important crop pests [22, 23]. Planthoppers generate vibrations primarily for mate localisation and courtship [24, 25], and their vibrational signals are remarkably consistent across taxa, with the exception of planthoppers in the family Delphacidae [25], at least some of which generate unusual vibrations using so-called ‘drumming’ organs [26]. Planthopper vibrations have previously been assumed to be generated by tymbal-like organs, homologous to those of cicadas [19, 27], or by the highly specialised delphacid ‘drumming’ organs [26, 28]. Yet morphological evidence from a range of planthopper taxa was lacking, and their vibration-generation mechanism was unknown. Here, we use a state-of-the-art morphological investigation of all 21 families of planthoppers (S1 Table) to study the vibration generation organs that are present throughout the group. We combine this analysis with experimental measurement of behavioural kinematics and the vibrations they produce to describe the remarkable mechanism of vibration generation in planthoppers and to explore the use of fast cyclical motions in the hidden world of substrate-borne vibrational communication.

## Results

### Snapping organ morphology

**Fig 1:**
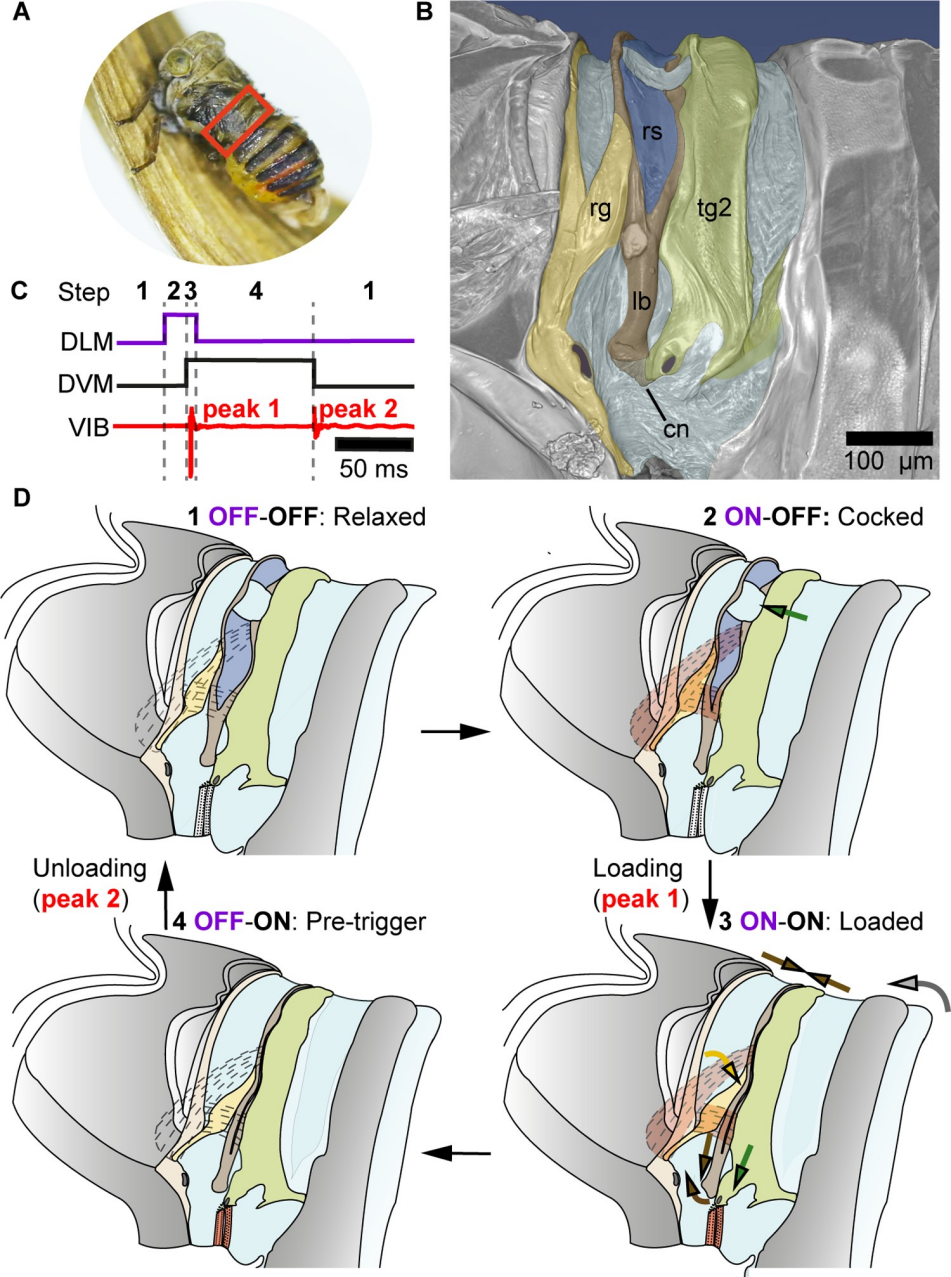
Vibration generation in planthoppers, using *A*. *bilobum* as a model. (A) Red box marks the snapping organ location. The forewings of this live specimen were removed to expose the snapping organ and its location on the abdomen. (B) False-colour SR-μCT scan of the snapping organ of *A*. *bilobum*, lateral view (scans deposited at CXIDB: http://cxidb.org/id-93.html). (C) Measured VIB for one sample recording and inferred activity of DLMs Idlm1-Idlm2 (purple) and DVMs IIedvm1-IIedvm2 (black) of the snapping organ during one cycle of vibration. (D) Schematic of the proposed four steps of the snapping organ required to generate one cycle of vibration. Muscles assumed to be in a relaxed state are transparent and labelled OFF, whereas those contracted are filled in red and labelled ON. Purple text refers to DLMs and black to DVMs. Loading and unloading result in the vibrational peaks seen in panel C. Structures and arrows colour-coded as follows: yellow, rg; brown, lb; light brown, cn (panel B only); dark blue: membrane with rs; green: tg2. Arrows indicate the direction of motion of these parts, whereas grey arrow denotes motion of abdomen. Latin numerals for muscles indicate segmental identity, whereas Arabic numerals indicate muscle set. cn, membranous connector; CXIDB, Coherent X-ray Imaging Data Bank; DLM, dorsal longitudinal muscle; DVM, dorsoventral muscle; edvm, external dorsoventral muscle; lb, Y-lobe; rg, ridge; rs, resilin; SR-μCT, synchrotron radiation microcomputed tomography; tg2, tergum 2; VIB, velocity of midabdomen in dorsoventral direction.

**Fig 2:**
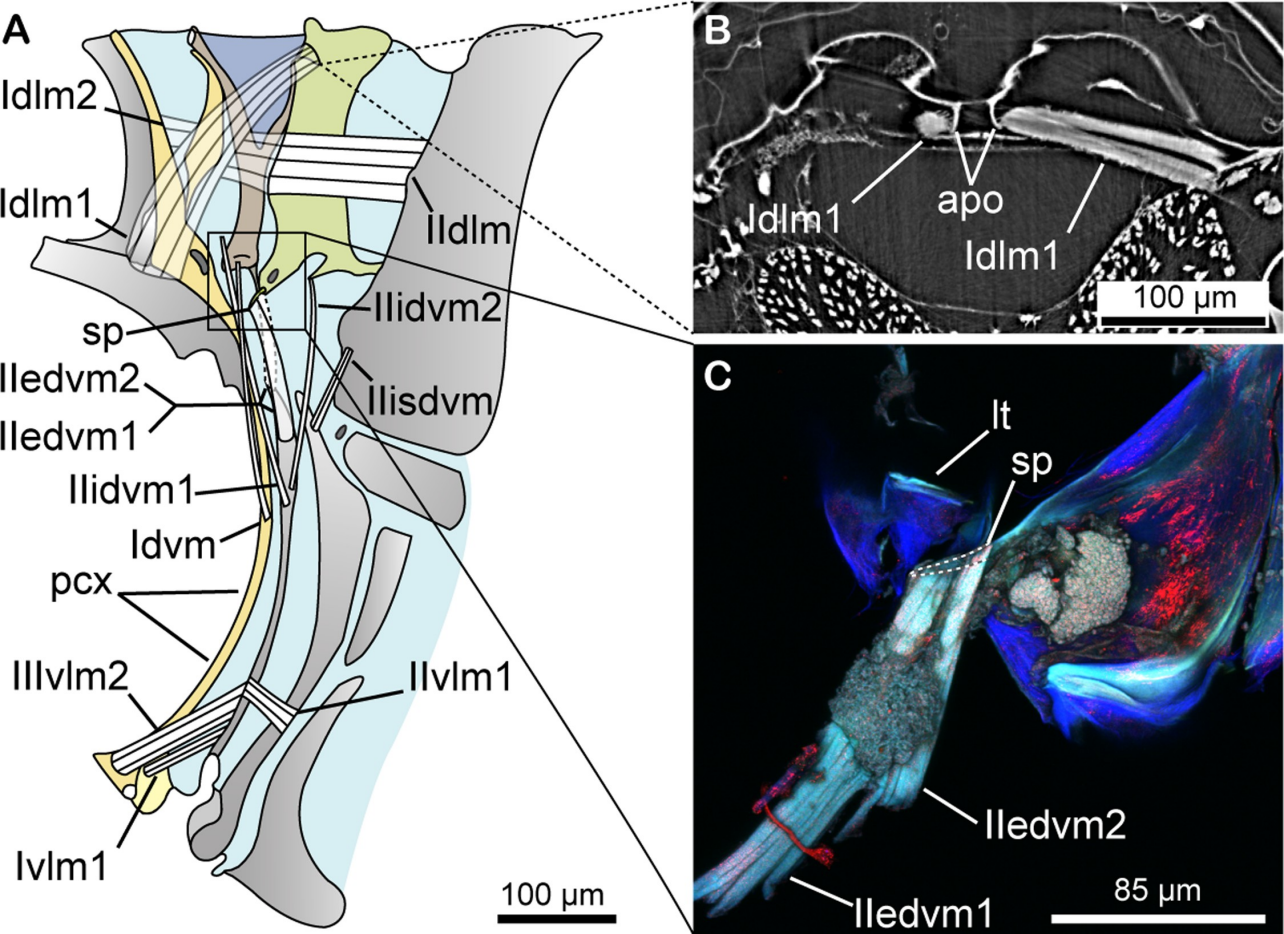
Generalized schematic of internal structure and musculature of the snapping organ. (A) Complete musculature of the first two abdominal segments. Square inset marks ventral junction of the lb base and tg2. (B) Transverse SR-μCT section of muscle-bearing apodeme of segment two, with hypertrophied Idlm1 inserting on it. (C) Confocal laser scanning microscopy image of lateral view of lb base-tg2 junction, with primary DVMs IIedvm1 and IIedvm2 inserting on sp (interrupted line). The angle of muscles IIedvm1 and IIedvm2 is somewhat distorted because of the fact that their ventral attachments have been severed. Colour coding of structures: yellow, rg; brown, lb; purple, rs membrane; green, tg2. Latin numerals for muscles indicate segmental identity, whereas Arabic numerals indicate muscle set. apo, apodeme of tergum 2; DLM, dorsal longitudinal muscle; DVM, dorsoventral muscle; edvm, external dorsoventral muscle; idvm, internal dorsoventral muscle; isdvm, intersegmental dorsoventral muscle; lb, Y-lobe; lt, list of base of Y-lobe; pcx, postcoxale; rg, ridge; rs, resilin; sp, spine of tergum 2; SR-μCT, synchrotron radiation microcomputed tomography; tg2, tergum 2; vlm, ventral longitudinal muscle.

We begin by characterising the morphology of the newly-described snapping organ in our model species, *Agalmatium bilobum* (Fulgoromorpha: Issidae). The snapping organ can be found dorsally on each side of the body at the junction between the metathorax and the abdomen, spanning the first two abdominal segments (Fig 1A and 1B and S1 Movie). The organ has a W shape; a ridge (Fig 1B) articulates at its base with the thorax (first ‘V’) and fuses at its tip to the anterior arm of a Y-lobe (Fig 1B), which has resilin (Fig 1B) between its arms (second ‘V’; Figs 1B and S1). The posterior arm of the Y-lobe is fused with the second segment, tergum 2 (tg2; Fig 1B) of the abdomen. The Y-lobe is linked at its base to an internal spine (sp; Fig 2C) of the second segment via a membranous connector (Figs 1B, 2A and 2C). Eight muscle pairs are directly associated with the snapping organ (Fig 2 and S2 Table and S1 Text), comprising three pairs of dorsal longitudinal muscles (DLMs) and five pairs of dorsoventral muscles (DVMs). Four other muscle pairs are indirectly associated with the snapping organ (ventral longitudinal muscles [vlms] IIIvlm2, Ivlm1, and IIvlm2 and intersegmental dorsoventral muscle [IIisdvm]) (Fig 2A). The snapping organ is not sexually dimorphic.

**Fig 3:**
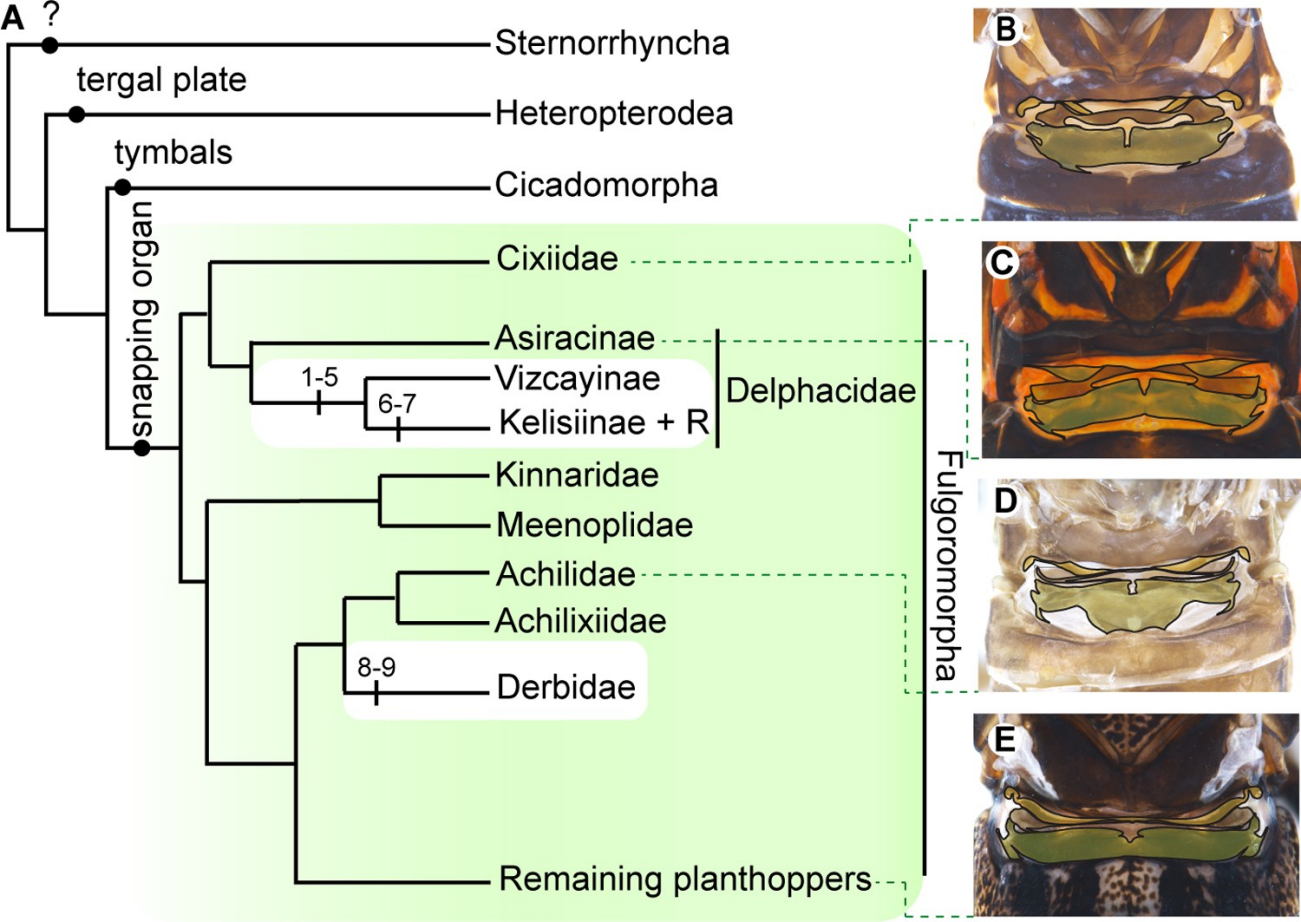
The snapping organ likely evolved once in the planthoppers. (A) Systematic distribution of the snapping organ (green, Fulgoromorpha) indicates a single origin at the root of planthopper phylogeny. White spaces within planthoppers indicate modification of snapping organs in the non-Asiracinae delphacids and the Derbidae. Numbers within the white spaces represent character states underlying the morphological transformations of the snapping organs in these planthoppers (see S4 Table). Other types of known abdominal vibrational organs are shown in the outgroups. Dorsal views (not to scale) of the snapping organs of (B) male *Pentastira* sp. (Cixiidae), (C) male *Asiraca clavicornis* (Delphacidae: Asiracinae), (D) male *Cixidia skaloula* (Achilidae), and (E) female *Caliscelis wallengreni* (Caliscelidae). Green dashed lines link snapping organs to their respective families; the branch of the tree labelled ‘remaining planthoppers’ also includes our model species *A*. *bilobum* (Issidae). Phylogenetic reconstruction is based on previous studies [29, 30]. R, remaining delphacid planthoppers.

Homologous vibrational organs are present throughout the entire planthopper clade (Fig 3 and S1 Table). The defining features of the musculature (Fig 2 and S2 and S3 Tables), innervation (S2 Fig), and external morphology (the ridge, Y-lobe, and connector) of the snapping organ are consistent and identifiable, despite variation in its proportions and shape across the planthoppers (Fig 3B–3E and S1 Text). Two groups deviate from this general picture: part of the family Delphacidae, in which the exoskeleton and musculature have been drastically reorganized to form an entirely different type of vibrational organ (S3 Fig and S1 Text), and part of Derbidae, which have an externally obscure snapping organ and also possess tentative stridulatory structures (Fig 3 and S4 Table). Based on their phylogenetic position, the deviations observed in these two groups are likely to be derived (Fig 3).

### Snapping organ biomechanics

**Fig 4:**
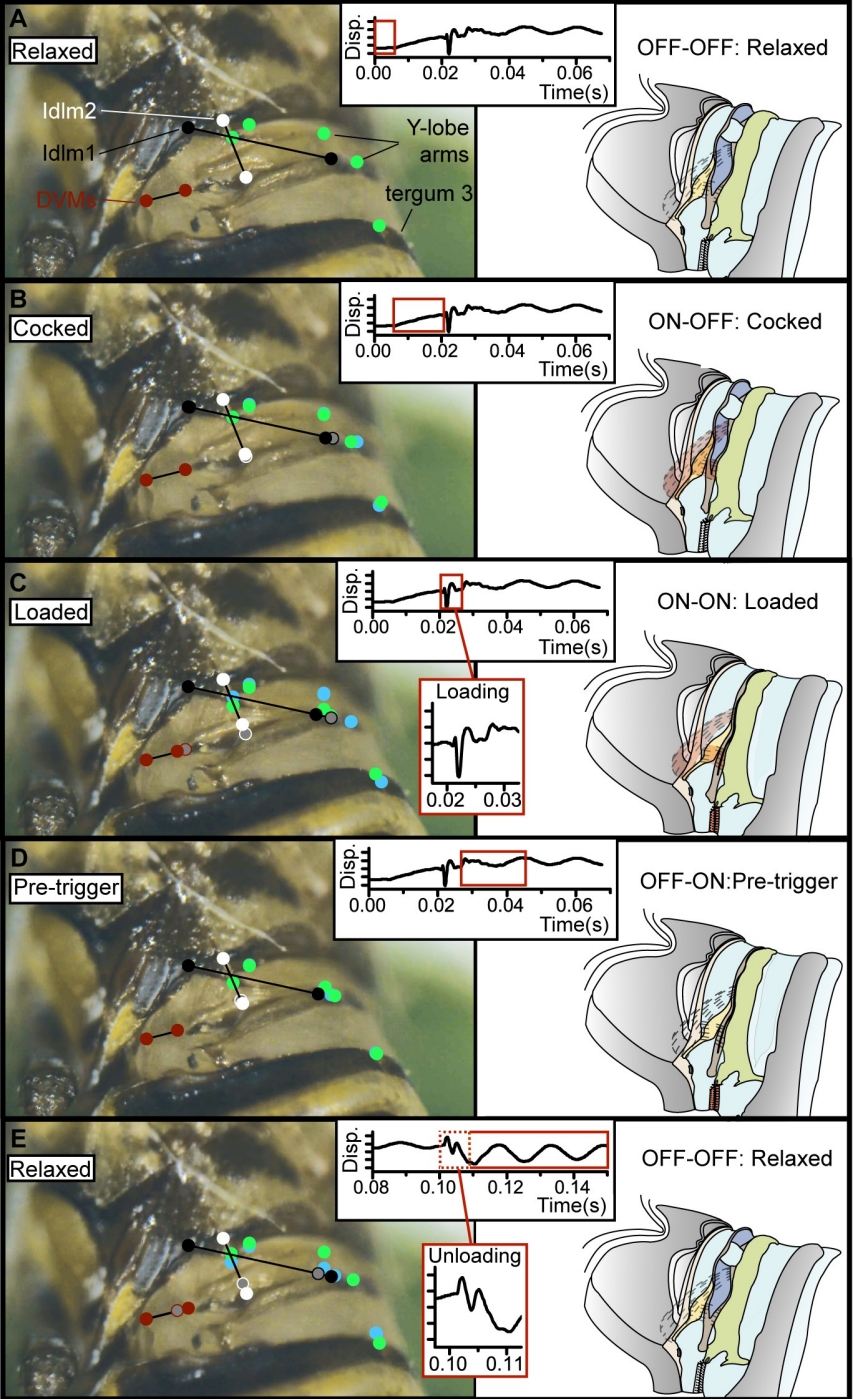
Stages of snapping organ mechanism in a male *A*. *bilobum*, illustrating external landmarks used to infer muscle strains internally (left) with corresponding stages of proposed mechanism shown diagrammatically (right). Muscle action was inferred from high-speed videography and laser vibrometry in conjunction with a separate microscopic study of the musculoskeletal anatomy to identify their origins and insertions (centre inset: Disp. of prothorax against time in seconds for one sample recording; identical axes on each panel): (A) relaxed, (B) cocked, (C) loaded, (D) pretrigger, (E) relaxed. (C) and (E) also have insets showing vibrometry recording for loading and unloading, respectively (Disp.–time). Origins and insertions of snapping organ muscles are symbolised by coloured circles (black: Idlm1; white: Idlm2; red: DVMs IIedvm1-IIedvm2). Muscle IIdlm is not included because the area it occupies does not undergo any noticeable change during stages (A)–(E) and is unlikely to contribute to the snapping organ mechanism. Unfilled coloured circles mark position of the respective muscle attachment in the previous panel; the change in distance between the points of muscle attachment indicates the extent of the muscle strain. Green and blue circles indicate position of other selected areas of the snapping organ in the current and previous panel, respectively. Red box on laser vibrometry inset panel indicates vibrational activity associated with the stage of motion represented in that panel. The underlying vibrometry data can be found within S1 Data. Disp., displacement; DLM, dorsal longitudinal muscle; DVM, dorsoventral muscle; edvm, external dorsoventral muscle.

To determine the kinematics of the snapping organ, we used high-speed videography and laser vibrometry on our model species, *A*. *bilobum* (Fig 4 and S1 Movie and S1 Data). Each vibrational cycle began with the snapping organ in its relaxed position (Figs 1D and 4A). Subsequently, the thorax/midabdomen was raised over a 15 ms timescale (Fig 4B). The first mechanical impulse followed (loading vibrational peak), which resulted in closed Y-lobe arms, extended ridge, and the base of the Y-lobe pulled down and rotated clockwise (Fig 4C). The system resonated in response, giving a jagged waveform over a 15–20 ms timescale (Fig 4D). The cycle was completed by a second mechanical impulse (unloading vibrational peak), in which the Y-lobe arms reopened, the ridge retracted, and the base of the Y-lobe rose and rotated back (Fig 4E). This resulted in whole-system resonance ultimately returning the organ to the same relaxed position as at the beginning of the cycle. Each vibration generation cycle takes place within 120 ms, and the mechanism does not generate any audible acoustic noise [27].

We propose that each cycle of vibration generation consists of four main steps (Figs 1D and 4). Transition from the relaxed state to the cocked state was comparatively slow (on a timescale of 15 ms), and the movements of landmarks on the external exoskeleton suggest that this phase of the cycle was driven directly by DLM contraction (Figs 2 and 4B). Whilst we do not have direct recordings of muscle activity, the distance between the origin and insertion points of both DLMs shortens at this point in the cycle (Fig 4B), and there is no other muscle whose action could produce this strain. The distance between these points shortens even further at the transition from the cocked state to the loaded state (Fig 4C), but this change occurs too quickly to be explained by direct muscle action alone. Specifically, the rate of change in the kinetic energy of the abdomen during loading implies energy release at a much higher power density than the DLMs and DVMs combined (Idlm1, Idlm2, IIedvm1, IIedvm2) could possibly supply through contraction (7,080 W kg^−1^, which is nearly 15 times the typical 500 W kg^−1^ power density for a muscle [31]; see S1 Methods and S1 Data). It follows that some form of elastic recoil, which acts as a kind of mechanical power amplifier, must be involved in the transition between the cocked and loaded states. This fast phase (0 to peak velocity taking 0.35 ms), which we term loading, is responsible for producing the first mechanical impulse transferring vibrational energy to the substrate. The distance between the origin and insertion points of the DVMs also shortens at this point in the cycle (Fig 4C), but contraction of these muscles alone cannot supply the mechanical energy at a high enough rate to explain the rapidity of the loading phase. Instead, the events at this transition are consistent with DVM contraction serving as an unlatching mechanism that triggers the rapid pulling down of the abdomen, followed by system resonance (Fig 4C).

The next phase of the cycle, in which the system transitioned to its pretrigger state, was a slow phase, probably involving muscle relaxation, over a 15–20 ms timescale. The subtle shift of exoskeleton positions, and particularly the lengthening of the distance between the points of origin and insertion of the DLMs (Fig 4D), is consistent with the DLMs relaxing during this phase. In contrast, the distance between the points of origin and insertion of the DVMs remain constant through this phase of the cycle, suggesting that they remain in their contracted state. The final transition in the cycle was from the pretrigger state to the relaxed state. This second fast phase, which we term unloading, is responsible for producing the second mechanical impulse transferring vibrational energy to the substrate. The associated increase in distance between the points of origin and insertion of the DVMs (Fig 4E) suggests that unloading is triggered by DVM relaxation, which causes the rapid return of the snapping organ to its relaxed conformation through a second release of stored elastic potential energy. There is no evidence for muscle contraction at this phase of the cycle, and we therefore infer that this elastic potential energy is likely to be stored in the deformed exoskeletal elements of the snapping organ.

**Fig 5:**
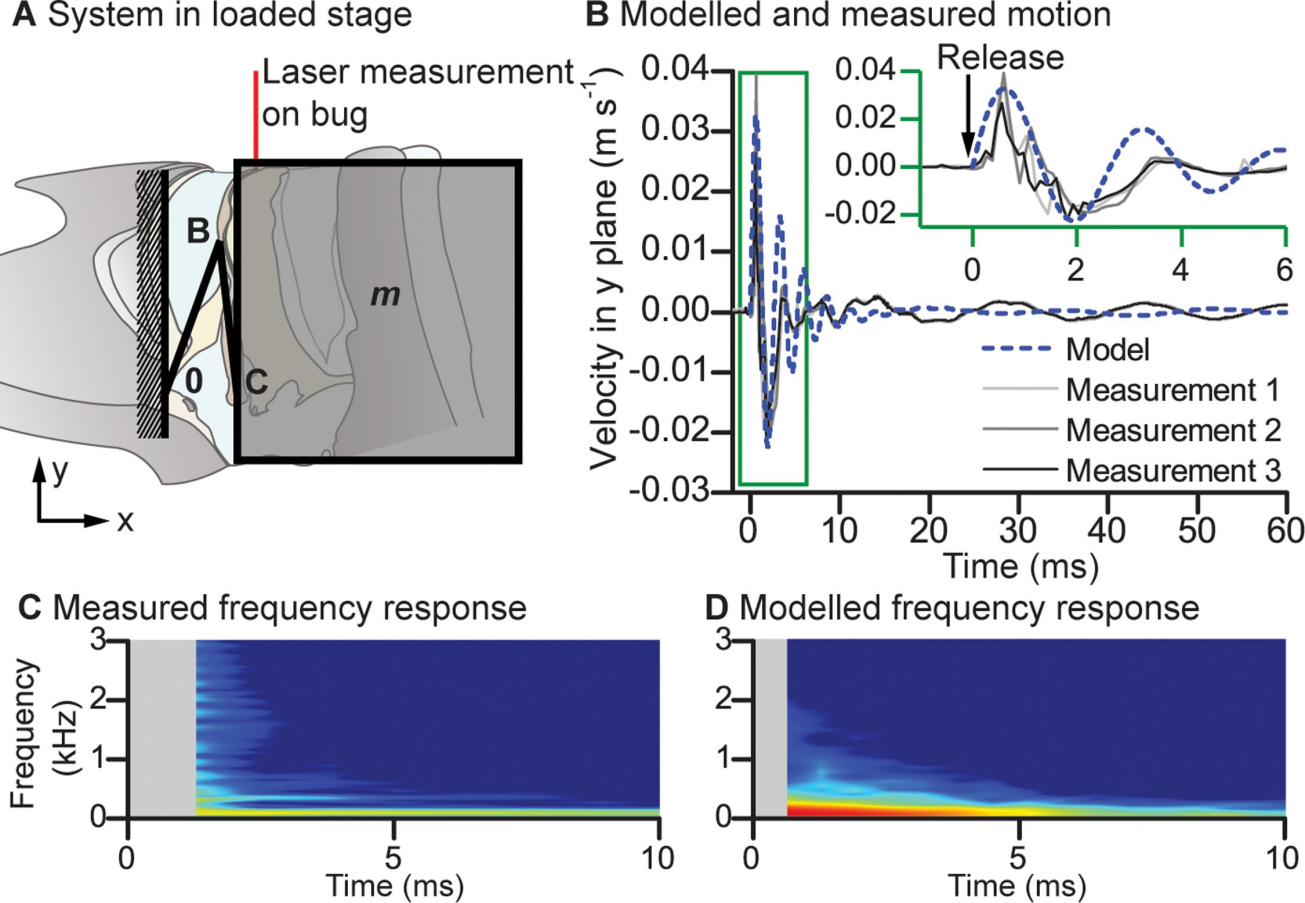
Modelled and measured motion of the snapping organ during unloading. (A) Schematic of the mathematical model and location of the laser vibrometry measurement in relation to the snapping organ. The model comprised two stiff beams in series representing the rg and anterior arm of the lb and could rotate at points 0 (junction of thorax and rg), B (junction of rg and lb), and C (base of lb). The thorax was fixed, but point C was connected to tg2 and the rest of the abdomen’s mass (*m*). Springs and damping elements not shown; see S4B Fig). Modelled (dashed blue line) and measured unloading motions in the dorsoventral direction (black, dark grey, and grey lines; measurements from the midabdomen of the same bug over three different cycles). The inset gives the same data over a shorter timescale, as indicated by the green box. (C and D) Frequency response from measured and modelled outputs, respectively, in which the colour scale gives relative magnitude in arbitrary units on an identical scale from low (blue) to high (red). The underlying data can be found within S1 Data. lb, Y-lobe; rg, ridge; tg2, tergum 2.

To verify whether passive release of elastic potential energy could be responsible for the fast unloading phase, we built a simplified mathematical model of the snapping organ, in which we replaced the ridge and the anterior arm of the Y-lobe with a pair of rigid bars connected in series to the thorax by a pair of torsional springs (Figs 5A and S4). The stiffness constants of these torsional springs were determined experimentally in a static loading experiment (S1 Methods). The abdomen and posterior arm of the Y-lobe were modelled as a mass-spring–damper system attached to the free end of the second rigid bar (Figs 5A and S4), and the spring constants and damping coefficients of this system were fitted as free parameters (S1 Methods). Quantitative comparison of the measured and modelled motion supports our supposition that the unloading phase can be explained through passive recoil of the Y-lobe, in which mechanical energy is stored elastically (Fig 5B and S1 Methods). When released, the elastic potential energy of these stiff springs acts to move the mass of the abdomen back to its relaxed state, resulting in resonant motion of the abdominal mass. More harmonic content is apparent in the measured vibrations than the modelled ones, which is not surprising given the simplicity of the model, but importantly from the perspective of information transfer, both the measured and the modelled spectra involve a broad range of different frequencies (Fig 5C and 5D).

### Snapping organ elastic recoil and transformation

The motion generated by the snapping organ during the two fast loading and unloading phases was on a timescale that would not have been possible through direct muscle action alone. The snapping organ instead uses two distinct elastic recoil mechanisms, each of which involves storing energy in springs, then releasing the stored energy quickly [8–11]. During the loading phase, the obvious candidate locations for elastic energy storage are the DLMs themselves, given that the exoskeleton itself deforms very little during loading (Fig 4B). This would mean that these muscles act both as engines, actively generating the force required for loading, and as springs, storing elastic energy within their deformed structure when subject to resistance against shortening from the exoskeleton. Muscles have previously been suggested to act as springs [10], and here the elastic energy storage is in the range achievable by the cross-bridges (energy density for paired Idlm1 and Idlm2 conservatively c. 2.47 J kg^−1^) [32]. We therefore suggest that resistance to shortening of the contracted DLMs allows these muscles to act as an elastic spring during the loading phase [31], storing energy slowly, then releasing this quickly when triggered. A latch must be involved to prevent early release of energy, and a mechanical constraint at the base of the Y-lobe could act as a latch that is removed when the DVMs contract, acting to trigger the release of elastic potential energy stored in the DLMs.

During the unloading phase, a more straightforward passive elastic recoil is the likely mechanism, as captured by our mathematical model (Fig 5). Energy is stored in stiff springs within the W-shaped exoskeleton linkage system that are deformed and therefore loaded during the loading phase (Fig 4C), but which return to their resting position and are therefore unloaded following the unloading phase (Fig 4E). The first elastic recoil event during the active loading phase thereby stores the energy that is released during the second elastic recoil event, which is the passive unloading phase. DVM relaxation is the likely trigger, with the membranous connector and acting as a possible cuticular latch preventing early release (Fig 2). Rapid recoil is made possible by DLM relaxation during the pretrigger step, and resilin between the Y-lobe arms (S1 Fig) will act to limit damage during recoil. Additional muscles may modulate the vibration during unloading (e.g., IIIvlm2), but the muscles are far too small to account for the power density during unloading (c. 765,000 W kg^−1^ if normalising the mechanical power by IIIvlm2 mass; Fig 2A and S1 Data).

In summary, the snapping organ uses two muscle contraction events per cycle and typically repeats its cycle every 0.3–1 s [33], giving a muscle contraction frequency of under 5 Hz (S5A Fig). In contrast, the frequencies of the mechanical impulses resulting from this motion as measured on the midabdomen were broadband under 3 kHz (shown for recoil in Fig 5C and 5D). Crucially, from a communication perspective, the complete system also acts to transfer mechanical motion from the snapping organ to the substrate. This represents another form of mechanical power transformation, albeit one that is modulated by the substrate. For motion vertical to the plant stem for one individual, the velocity ratio of motion measured on the plant relative to motion measured on the insect midabdomen indicates that the velocity of motion is attenuated by 83% (average −15.5 ± 6.2 dB), with lower attenuation in velocity of motion between the prothorax and plant at 71% attenuation (average −10.5 ± 5.5 dB, S1 Data and S5 Fig).

## Discussion

The consistency of the snapping organ’s morphology, and its systematic distribution across planthoppers indicates that this most likely represents a conserved mechanism for generating abdominal vibrations across the Fulgoromorpha. Previous studies have only examined delphacid vibrational organs [24, 26, 34], but our analysis of their peculiar morphology indicates that the drumming organs of delphacids are the exception and not the rule. The consistency of snapping organ morphology across the rest of the planthoppers provides a clear mechanistic explanation for the observed uniformity of their vibrational signals [25, 33]. These findings reflect the fundamental importance of vibrational signals in planthopper communication.

The functional morphology of the snapping organ also reveals some remarkable functional convergences and some equally remarkable mechanistic differences between the mechanical communication mechanisms of planthoppers and their close relatives, the cicadas [12, 24]. Both make use of paired elastic recoil mechanisms and low-frequency active muscle contractions to enhance the efficiency and efficacy of communication, using exoskeletal integration to transform mechanical impulses into substrate vibration [2, 12]. Driven by a single muscle, the cicadas’ tymbal organs use buckling instability of multiple stiff ribs to store and release elastic energy, turning slow muscle contraction into fast motion as the ribs buckle [12]. Muscle relaxation and the release of energy stored in resilin pads causes the ribs to restraighten again, leading to a second step involving elastic energy release [15]. In contrast, the snapping organ uses two different energy-storage mechanisms for paired elastic recoil: elastic storage in contracted muscle for loading and elastic storage in the deformed exoskeleton for unloading. Instead of buckling like the ribs of a tymbal, the arms of the Y-lobe in the snapping organ use snapping motions similar to those used in fast raptorial strikes by jaws and claws [8, 9]. Finally, whereas tymbal vibrations in most cicadas are often associated with resonant chambers that act to transform motion into loud acoustic signals [12], the snapping organ is specialised for substrate-borne vibration generation, with comparable muscle contraction rates that act to transfer mechanical energy into vibrations of the substrate [12].

In conclusion, the unique biomechanics of the snapping organ demonstrate the general importance of elastic recoil mechanisms in the fast motions of small arthropods, extending our knowledge of such mechanisms beyond the simpler one-off ballistic motions that characterise jumping, predatory strikes, and feeding. Elastic recoil is a very general mechanism allowing small animals to overcome the limitations of their size and enabling robust vibrational communication.

## Materials and methods

### Insects

Individuals of *A*. *bilobum*, the model planthopper species used in this study, were collected in large numbers (*n* = 250) in late April 2017 as fourth/fifth-instar larvae or adults from Lycabettus Hill, Athens, Greece, and were imported to Oxford, UK under DEFRA Plant Health Licence no. 52972/198417/6. Larvae were reared into adulthood in mesh cages (47.5 cm × 47.5 cm × 47.5 cm) kept at 22–29°C, 50% humidity, with a 16:8 photoperiod (light/dark).

In addition, the morphology of specimens from more than 130 taxa were examined, covering the entire phylogenetic spectrum of Fulgoromorpha. S1 Table details the techniques used to examine the morphology of the snapping organ for each species, along with its preservation method.

### Morphological analysis

Planthoppers belonging to 12 families (including three specimens of *A*. *bilobum*: adult male, female, and larva) were used for synchrotron radiation microcomputed tomography (SR-μCT) at the TOMCAT beamline, Swiss Light Source (SLS), Paul Scherrer Institute, Switzerland (S1 Table). All specimens were scanned at a beam energy of 15.99 keV with a final pixel size of 1.625 μm, allowing visualisation of even the smallest muscles and nerves of the snapping organ (Figs 1B and 2B and S2 and S3B–S3D), which were otherwise not detected by other techniques. Three-dimensional reconstruction was carried out using Amira 6.1 software (Mercury Systems, Andover, MA, USA). All shown tomographic data (reconstructed TIFFs) for the two imaged species (*A*. *bilobum* and *Stenocranus minutus*) are freely available at CXIDB (http://cxidb.org/id-93.html) [35]. Colouration and labelling of figures were performed in Adobe Illustrator CS6. In order to reveal the primary DVMs operating the snapping organ in *A*. *bilobum*, the ventral junction between the Y-lobe and tg2 were excised from an ethanol-preserved (70%) male (Fig 2C). The dissected sample was placed between two cover slips in 70% ethanol and was imaged with a laser confocal scanning microscope (Olympus FV1000; Olympus, Tokyo, Japan) at a laser wavelength of 488 nm. The morphologies of specimens belonging to all 21 planthopper families were examined under light microscopy. Images of the snapping organ of four species of planthoppers shown in Fig 3 were taken using a Leica M165c microscope equipped with a Leica DFC490 camera (Leica, Wetzlar, Germany). The final, stacked images were combined using Helicon Focus (Helicon Soft, Kharkiv, Ukraine). Image brightness adjustment was performed in Adobe Photoshop, and drawings were generated in Adobe Illustrator CS6.

### Laser Doppler vibrometry

To record vibrational signals, planthoppers were placed on a dried grass (*Schedonorus giganteus*) stem (17 cm in height). The base of the stem was inserted inside an empty c. 1-cm–diameter tube and was held in place by aluminium foil. Vibrational signals were recorded by a laser Doppler vibrometer (Polytec PDV-100; Polytec, Waldbronn, Germany), focussed at different positions approximately orthogonal to the stem and bug. A sampling frequency of 9.6 kHz was used for recordings at a gain of 100 mm/s/V. Recording started immediately once the planthoppers were placed on the stem. Each recording lasted 6 minutes and was repeated until the animal either ended its vibrational call or after four recordings if no songs were present.

A total of 61 recordings were made, 31 on single planthoppers, 26 on male–female groups, and four on male–male groups, using a total of 19 individuals (12 males, 7 females). Recordings from two individuals are included in S1 Data, in which the laser was focussed on the plant stem (individual 1), bug prothorax (individual 1), bug genitalia (individual 2), or bug midabdomen (individual 1). All vibrometry recordings were similar in the type and pattern of motion observed, so the data presented in S1 Data and S5 Fig are assumed to be representative. Attenuation of motion during loading and unloading from the midabdomen to the plant stem and prothorax to the plant stem was calculated in decibels (S1 Data). Vibrometry figures were drawn using Raven Lite 2.0 (Cornell Lab of Ornithology, Ithaca, NY, USA) and OriginPro 8. To stimulate vibration generation, we used playback tracks of recorded songs. The stem was vibrated 7.3 cm from the base by a pin glued to a small piezo disc (RS Components, Corby, UK), which was glued on an inverted plastic cup. Playback songs consisted of prerecorded and amplified vibrational signals of both sexes. All males responded to the playback by emitting a series of pulses for several minutes.

### High-speed video recordings

The motion of the snapping organ in *A*. *bilobum* was captured with a high-speed camera (Grasshopper3 2.3 MP Colour USB3 Vision, Sony Pregius IMX174; Point Grey, Richmond, BC, Canada) mounted on a Leica S8 AP0 stereomicroscope, recording at a rate of 100 frames s^−1^. Videos were recorded directly to a computer using Spinnaker SDK-1.3.0.21 software (Point Grey). A total of three males were video recorded, and a movie and still frames from one male are given in Fig 4 and S1 Movie. The males were filmed over multiple cycles, frames were classified into the different stages of the mechanism, and the clearest frames were chosen from these classified groups within Fig 4. Pixel coordinates of three points on the bug prothorax were quantified for each frame used in Fig 4 to check for alignment of the bug within the video frame over time. Standard deviation over the five frames for each of the three points was within the order of 0.01 pixels, suggesting the bug has limited movement within the video frame over successive cycles (also supported by S1 Movie). Prior to recording, it was necessary to expose the snapping organ by removing the fore and hind wings with a scalpel. The males were then left on their host plant for one hour to recover after wing removal before playback recordings were started to stimulate vibration generation. Based on our observations, the motion captured in S1 Movie is representative of the vibration-generation mechanism across different individuals.

### Calculations and modelling

The vibrometry recordings were analysed to calculate the peak energy and power of the loading and unloading motions (S1 Data). Maximum and minimum peak velocities and the timings of the peaks were extracted from the vibrometry data. The peak kinetic energy of the motion was calculated from the speed of the measured dorsoventral translation of the abdominal mass, and the corresponding mechanical power was determined by dividing this peak kinetic energy by the time taken to reach it from rest. The muscle power density that would be required to generate this motion through direct muscle contraction was calculated by dividing these values by the relevant muscle mass, as measured from SR-μCT measurements of *A*. *bilobum*, modelling muscles as cylinders with a density of 1,060 kg m^−3^ [36].

A mathematical model was developed to support the interpretation that unloading was due to elastic recoil of the system (Figs 5 and S4). The model included the abdomen as a mass attached to two rigid bars in series (anterior Y-lobe arm and ridge, respectively), each with a stiff rotational spring at their junctions. The anterior bar was fixed to a surface, representing the thorax. Springs and dampers acting on the mass of the abdomen modelled the combined action of the muscles, resilin, other exoskeletal components, and interior morphology on the motion of the mass in the dorso–ventral and anterior–posterior planes. Full details of the model are given in S1 Methods.

## Supporting information

S1 Fig Dissected snapping organ of a male *A*. *bilobum*. (A) Bug viewed under light microscopy; (B) bug excited by UV light, the externally visible fluorescence indicating the presence of rs on the membrane between the arms of the lb (arrowed). Dashed arrow indicates other areas of fluorescence on the abdomen that are not consistent between specimens. rs whose presence is revealed by fluorescence on the metathorax is unlikely to participate in the snapping organ mechanism. lb, Y-lobe; rs, resilin; UV, ultraviolet
(TIF)

S2 Fig Abdominal nervous system of a generalised planthopper. Nervous system reconstructed from SR-μCT of *A*. *bilobum* and an unidentified nogodinid and fulgorid (S1 Table), whose gross morphology of the nervous system was similar. The muscles of the second abdominal segment (top right) are innervated from the second abdominal nerve. Muscles from the first abdominal segment are innervated from their corresponding nerve. Innervation for muscles IIidvm1-IIisdvm could not be traced. idvm, internal dorsoventral muscle; isdvm, intersegmental dorsoventral muscle; msg, mesothoracic ganglion; n. ab. 1, abdominal nerve of segment 1; n. ab. 2, abdominal nerve of segment 2; n. ab. 3, abdominal nerve of segment three; n. ab. 4–9, abdominal nerve of segments four to nine; n. mt., metathoracic nerves; SR-μCT, synchrotron radiation microcomputed tomography
(EPS)

S3 Fig Drumming organ of a generalized non-asiracine male delphacid, *S*. *minutus*. (A) Dorsal view of drumming organ in relaxed conformation in an ethanol-preserved specimen. (B) False-colour SR-μCT volume rendered image of the drumming organ. The top part of the organ is virtually sliced off, revealing the attachments of muscle Idlm1. (C) Lateral view of the drumming organ. (D) The same image, virtually made transparent to show the DVMs operating the drumming organ and their attachments. Dashed lines show the boundaries of the exoskeletal components of the drumming organ. Colour coding of structures: yellow = rg; brown = modified lb; green = tg2; pink = tg1. Tomographic data for this species are freely available at CXIDB: http://cxidb.org/id-93.html. cp, central plate; DLM, dorsal longitudinal muscle; DVM, dorsoventral muscle; lb, Y-lobe; lt, transverse list of modified Y-lobe; rg, ridge; SR-μCT, synchrotron radiation microcomputed tomography; tg1, tergum one; tg2, tergum 2
(TIF)

S4 Fig Mathematical model of snapping organ. Two rigid bars articulate at points 0, *B*, and *C*, as dictated by torsion springs *k*_*3*_ and *k*_*4*_. The first rigid bar is attached to a fixed surface at 0, and a lumped mass (*m*) is attached to the second rigid bar at *B*. A system of linear springs and dampers connects to the mass at *B*. All parameters are measured from the real system (see S1 Methods), with the exception of *k*_*1*_, *k*_*2*_, *λ*_*1*_, and *λ*_*2*_, which were fitted by eye to match the measured motion (Fig 5). The model starts in the loaded state and then moves to the relaxed state; thus, unloading is modelled.
(TIF)

S5 Fig Vibrometry recordings of vibration generation in a male *A*. *bilobum*, in which black, grey, and light grey represent recordings from the midabdomen, prothorax, and plant stem, respectively. Three repeats are plotted on each panel. (A) Time–velocity plots of recordings across multiple cycles, labelling the location of L and U phases over time. (B) Data from panel A at higher temporal resolution for L, aligned in the time axis by the first peak maximum amplitude. (C) Data from panel A at higher temporal resolution for U, aligned in the time axis by the first peak maximum amplitude. Sample rates were 9.6 kHz, and all data are from the same individual. Data also shown in S1 Data, including attenuation calculations for minimum and maximum peaks during loading and unloading. L, loading; U, unloading
(EPS)

S1 Table Species list of examined taxa, along with data on individual type of preservation, observation method, and depository. Examination of dry mounted specimens using microscopy only allowed documentation of exoskeletal morphology, while musculature was also studied in ethanol-preserved specimens. Use of SR-μCT permitted examination of the exoskeleton, musculature, and innervation of the snapping organ. Illustrations from the literature allowed examination of the external morphology of the vibrational organs of certain delphacids. The abovementioned observation methods allowed us to document the presence of a snapping organ (based on its defining characters) in all examined taxa, with the exception of non-Asiracinae delphacids, the latter having modified snapping organs. BMNH, Natural History Museum, London; DPC, Davranoglou Private Collection; MMBC, Moravian Museum, Brno; OUMNH, Oxford University Museum of Natural History; SR-μCT, synchrotron radiation microcomputed tomography; UG, University of Gdansk
(DOCX)

S2 Table Muscles operating the snapping organ of Fulgoromorpha, based on dissection of ethanol-preserved specimens and SR-μCT scans. Function of muscles was inferred by high-speed videography, power calculations of laser Doppler vibrometry recordings of *A*. *bilobum*, and artificial contraction of the respective muscles using a pair of forceps in ethanol-preserved specimens. SR-μCT, synchrotron radiation microcomputed tomography
(DOCX)

S3 Table List of previous names for planthopper muscles (all delphacids), homologised with the terminology applied in the present study for the snapping organ musculature. Inferences of homology and segmental identity were based on muscle innervation and location from SR-μCT and ethanol-preserved specimens. En-dash (–) denotes that the character is either absent or not reported by the study in question. SR-μCT, synchrotron radiation microcomputed tomography
(DOCX)

S4 Table Character states representing major transformations of the snapping organ in Fulgoromorpha. Characters of delphacids largely based on the meta-analysis of Asche, 1990 [34] and our own observations of ethanol-preserved and SR-μCT specimens. Order of character states does not imply evolutionary sequence. SR-μCT, synchrotron radiation microcomputed tomography
(DOCX)

S1 Data Three tabs show (i) vibrometry data from the midabdomen during loading and unloading (three measurements from individual 1), along with calculations of peak coordinates, time since x-axis crossing, and attenuation of peak motions from midabdomen to plant substrate and prothorax to plant substrate; (ii) vibrometry data from the laser focussed on plant substrate (individual 1), prothorax (individual 1), and bug genitalia (individual 2) during loading and unloading; and (iii) power calculations.
(XLSX)

S1 Movie High-speed camera recordings (100 frames per second) of the snapping organ of a male *A*. *bilobum* in action. 0–10 s, lateral view; 10–15 s, caudal view; 15–20 s, dorsal view.
(MP4)

S1 Methods Gives additional detail for the methods employed, including detail on the insects, morphological analysis, power calculations, and mathematical model. (DOCX)

S1 Text Gives an extended description of the snapping organ in our model species *A*. *bilobum* (Issidae), outlines the evidence of the presence of the snapping organ in other planthopper species, and gives an in-depth description of the delphacid ‘drumming organ’. (DOCX)

## Abbreviations

apo: apodeme of tergum 2
cn: membranous connector
CXIDB: Coherent X-ray Imaging Data Bank
Disp.: displacement
DLM: dorsal longitudinal muscle
DVM: dorsoventral muscle
edvm: external dorsoventral muscle
idvm: internal dorsoventral muscle
isdvm: intersegmental dorsoventral muscle
lb: Y-lobe
lt: list of base of Y-lobe
pcx: postcoxale
R: remaining delphacid planthoppers
rg: ridge
rs: resilin
SLS: Swiss Light Source
sp: spine of tergum 2
SR-μCT: synchrotron radiation microcomputed tomography
tg2: tergum 2
VIB: velocity of midabdomen in dorsoventral direction
vlm: ventral longitudinal muscle
