# Correction: Planthopper bugs use a fast, cyclic elastic recoil mechanism for effective vibrational communication at small body size

**DOI:** 10.1371/journal.pbio.3001047

**Published:** 2020-12-09

**Authors:** Leonidas-Romanos Davranoglou, Alice Cicirello, Graham K. Taylor, Beth Mortimer

The authors identified a scaling issue with some of the measurements in the manuscript.

1. The length of the scale bar overlain on the microcomputed tomography (μCT) data in Fig 1B was stated incorrectly, and should have read 200μm rather than 100μm. Please see corrected Fig 1.
2. During investigation of this error, the authors realized that the μCT measurements of muscle length and width that they had used to estimate muscle volume had also been scaled incorrectly in S1 Data. In correcting these measurements, the authors have replaced the cylinder approximations of muscle volume with exact volumetric measurements of the muscles made directly from the μCT data. The effect of this correction is to increase the estimate of the combined mass of the dorsal longitudinal muscles (DLMs) Idlm1 and Idlm2, and dorso-ventral muscles (DVMs) IIedvm1 and IIedvm2 by 3.4%. The authors have added the corresponding muscle segmentations to the tomographic data that is freely available at CXIDB (http://cxidb.org/id-93.html). Please see corrected S1 Data.
3. This change in the estimate of muscle mass has a corresponding effect on the specific power and energy calculations.
  The 5^th^ sentence in paragraph 2 of the subsection of the Results titled, “Snapping organ biomechanics,” corrected text should read: “Specifically, the rate of change in the kinetic energy of the abdomen during loading implies energy release at a much higher power output than the DLMs and DVMs combined (Idlm1, Idlm2, IIedvm1, IIedvm2) could possibly supply through contraction (implied specific power: 7 kW kg^−1^, which is an order of magnitude higher than the maximum 500 W kg^−1^ specific power output reported for muscle [31]; see S1 Methods and S1 Data).”
  The fifth sentence in the first paragraph of the subsection of the Results titled “Snapping organ elastic recoil and transformation” should read: “Muscles have previously been suggested to act as springs [10], and here the elastic energy storage is in the range achievable by the muscle cross-bridges [32] if all of the elastic energy were stored in the DLMs (implied specific energy for Idlm1 and Idlm2: 2.63 J kg^−1^).”
  The last sentence in the second paragraph of the same subsection should read: “Additional muscles may modulate the vibration during unloading (i.e., IIIvlm2), but even the largest of these is an order of magnitude too small to account for the rate of energy release during unloading (implied specific power: 5 kW kg^−1^ if normalising mechanical power by IIIvlm2 mass; Fig. 2A and S1 Data).”
4. In rechecking all of the numbers that they had reported, the authors saw that the values of the muscle spring stiffnesses k3 and k4 assumed in the mathematical model were written incorrectly in S1 Methods. These should have been written as 0.31 N μm rad^-1^ and 0.62 N μm rad^-1^ respectively. The mathematical model and its results are unchanged. Please see corrected S1 Methods.

**Fig 1:**
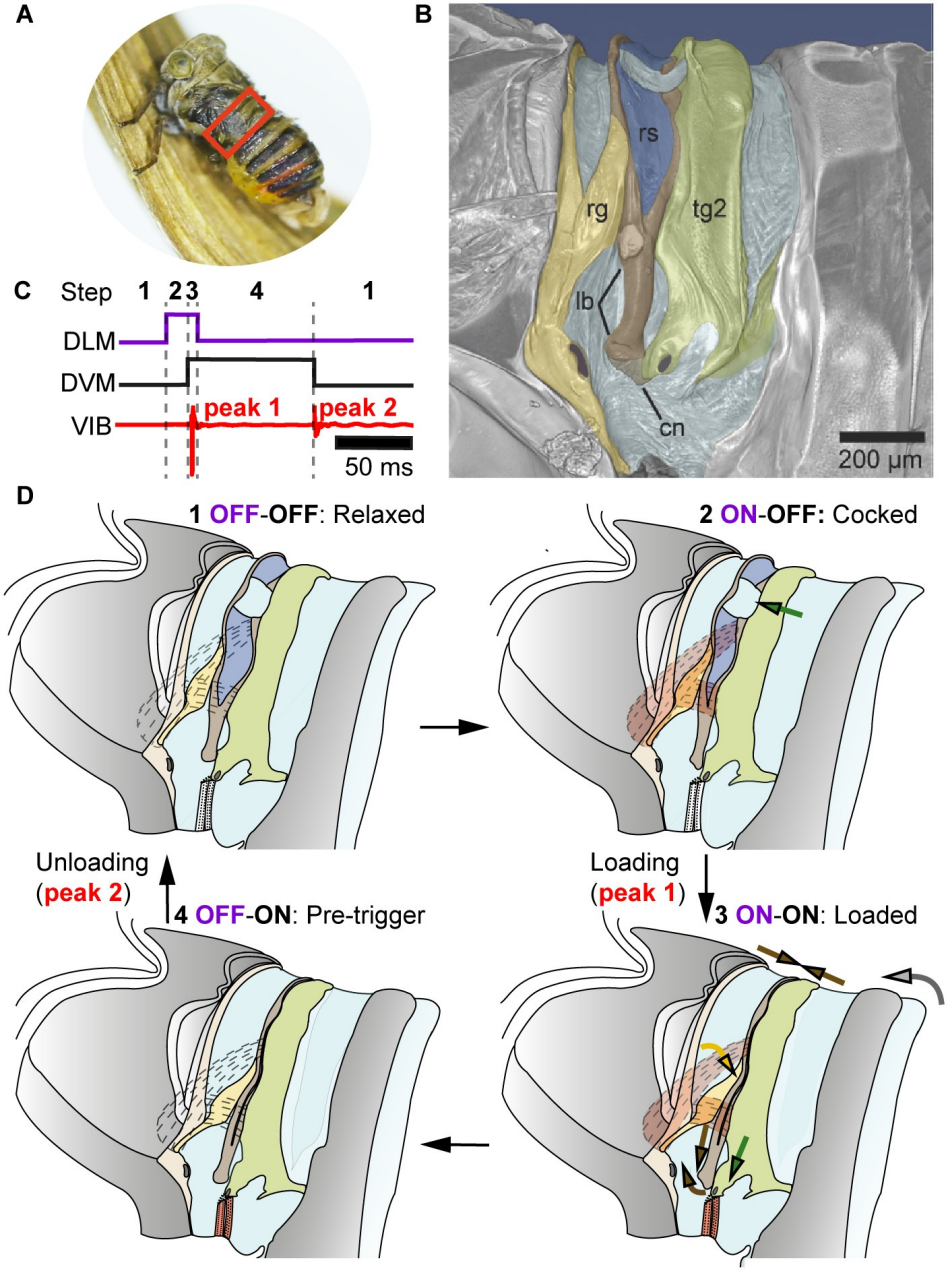
Vibration generation in planthoppers, using A. bilobum as a model. (A) Red box marks the snapping organ location. The forewings of this live specimen were removed to expose the snapping organ and its location on the abdomen. (B) False-colour SR-μCT scan of the snapping organ of A. bilobum, lateral view (scans deposited at CXIDB: http://cxidb.org/id-93.html). (C) Measured VIB for one sample recording and inferred activity of DLMs Idlm1-Idlm2 (purple) and DVMs IIedvm1-IIedvm2 (black) of the snapping organ during one cycle of vibration. (D) Schematic of the proposed four steps of the snapping organ required to generate one cycle of vibration. Muscles assumed to be in a relaxed state are transparent and labelled OFF, whereas those contracted are filled in red and labelled ON. Purple text refers to DLMs and black to DVMs. Loading and unloading result in the vibrational peaks seen in panel C. Structures and arrows colour-coded as follows: yellow, rg; brown, lb; light brown, cn (panel B only); dark blue: membrane with rs; green: tg2. Arrows indicate the direction of motion of these parts, whereas grey arrow denotes motion of abdomen. Latin numerals for muscles indicate segmental identity, whereas Arabic numerals indicate muscle set. cn, membranous connector; CXIDB, Coherent X-ray Imaging Data Bank; DLM, dorsal longitudinal muscle; DVM, dorsoventral muscle; edvm, external dorsoventral muscle; lb, Y-lobe; rg, ridge; rs, resilin; SR-μCT, synchrotron radiation microcomputed tomography; tg2, tergum 2; VIB, velocity of midabdomen in dorsoventral direction.

## Supporting information

S1 Data Three tabs show (i) vibrometry data from the midabdomen during loading and unloading (three measurements from individual 1), along with calculations of peak coordinates, time since x-axis crossing, and attenuation of peak motions from midabdomen to plant substrate and prothorax to plant substrate; (ii) vibrometry data from the laser focussed on plant substrate (individual 1), prothorax (individual 1), and bug genitalia (individual 2) during loading and unloading; and (iii) power calculations. (XLSX)

S1 Methods Gives additional detail for the methods employed, including detail on the insects, morphological analysis, power calculations, and mathematical model. (DOCX)
