# Supplementary figures and images for: Planthopper bugs use a fast, cyclic elastic recoil mechanism for effective vibrational communication at small body size
Source: PLoS Biol. 2019 Mar 12;17(3):e3000155. doi: 10.1371/journal.pbio.3000155 (PMC6413918; doi:10.1371/journal.pbio.3000155)

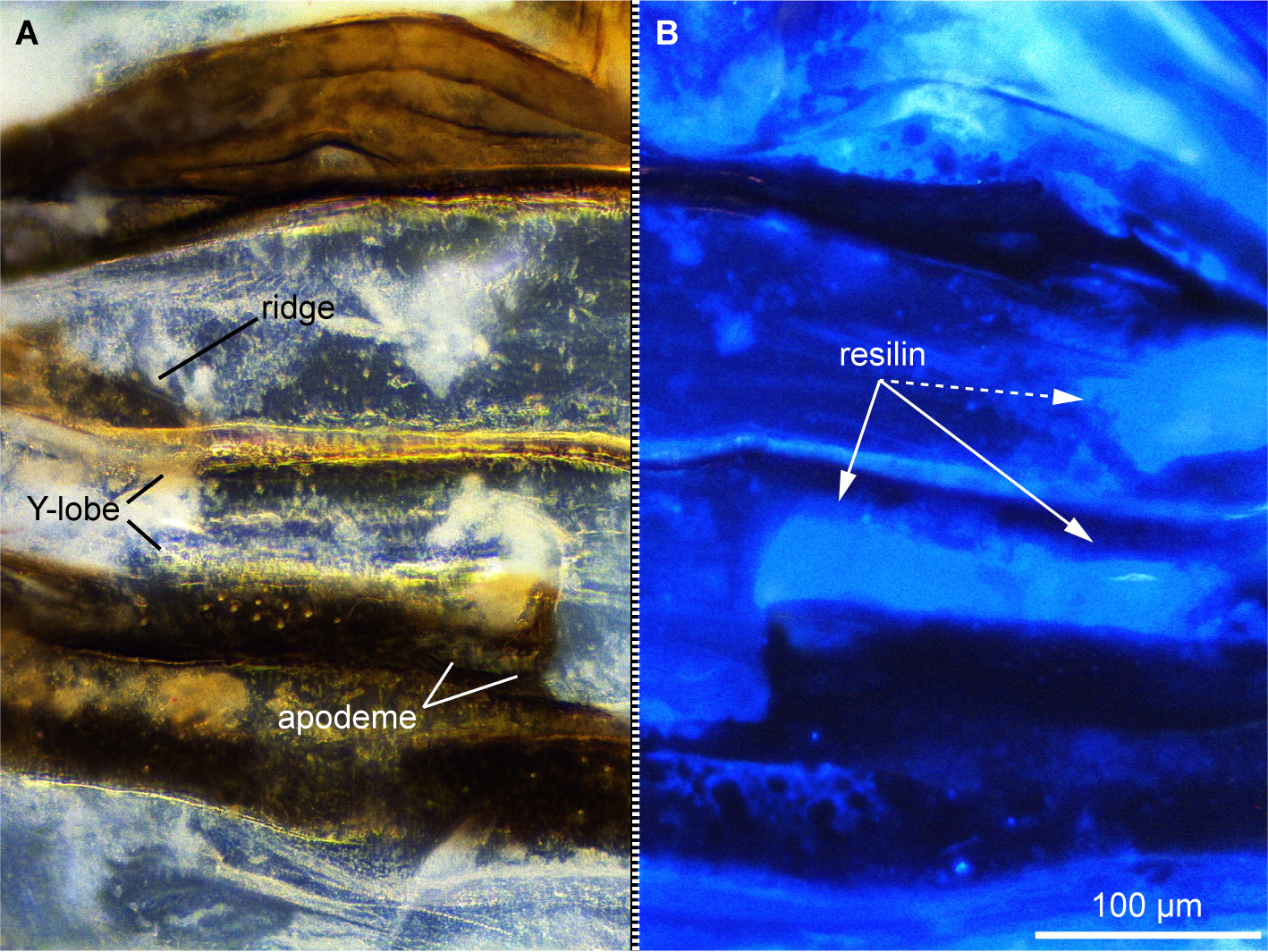

Supplement: S1 Fig — (A) Bug viewed under light microscopy; (B) bug excited by UV light, the externally visible fluorescence indicating the presence of rs on the membrane between the arms of the lb (arrowed). Dashed arrow indicates other areas of fluorescence on the abdomen that are not consistent between specimens. rs whose presence is revealed by fluorescence on the metathorax is unlikely to participate in the snapping organ mechanism. lb, Y-lobe; rs, resilin; UV, ultraviolet (TIF) [file pbio.3000155.s001.tif]

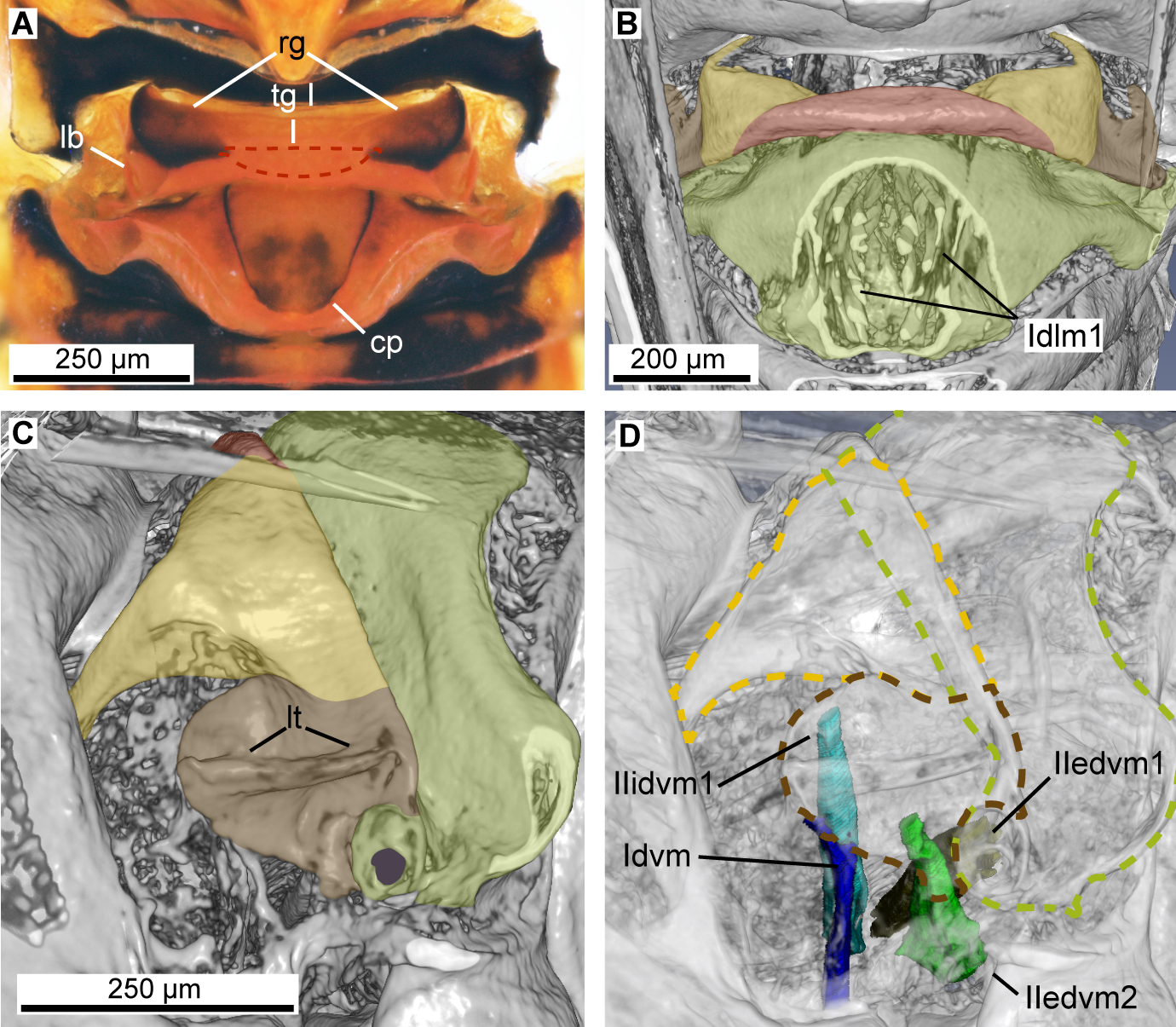

Supplement: S3 Fig — (A) Dorsal view of drumming organ in relaxed conformation in an ethanol-preserved specimen. (B) False-colour SR-μCT volume rendered image of the drumming organ. The top part of the organ is virtually sliced off, revealing the attachments of muscle Idlm1. (C) Lateral view of the drumming organ. (D) The same image, virtually made transparent to show the DVMs operating the drumming organ and their attachments. Dashed lines show the boundaries of the exoskeletal components of the drumming organ. Colour coding of structures: yellow = rg; brown = modified lb; green = tg2; pink = tg1. Tomographic data for this species are freely available at CXIDB: http://cxidb.org/id-93.html. cp, central plate; DLM, dorsal longitudinal muscle; DVM, dorsoventral muscle; lb, Y-lobe; lt, transverse list of modified Y-lobe; rg, ridge; SR-μCT, synchrotron radiation microcomputed tomography; tg1, tergum one; tg2, tergum 2 (TIF) [file pbio.3000155.s003.tif]

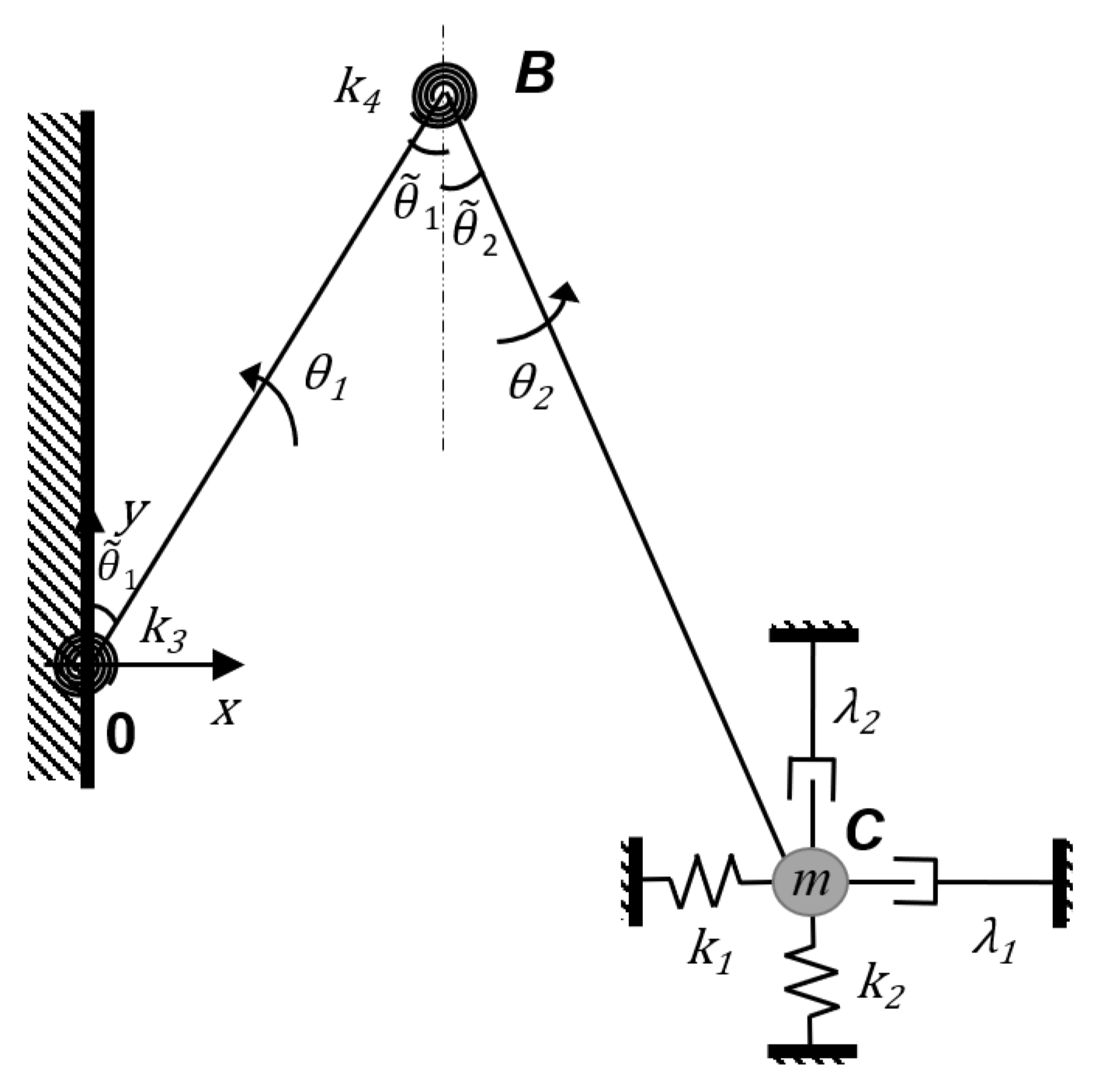

Supplement: S4 Fig — Two rigid bars articulate at points 0, B, and C, as dictated by torsion springs k3 and k4. The first rigid bar is attached to a fixed surface at 0, and a lumped mass (m) is attached to the second rigid bar at B. A system of linear springs and dampers connects to the mass at B. All parameters are measured from the real system (see S1 Methods), with the exception of k1, k2, λ1, and λ2, which were fitted by eye to match the measured motion (Fig 5). The model starts in the loaded state and then moves to the relaxed state; thus, unloading is modelled. (TIF) [file pbio.3000155.s004.tif]
