# Supplementary material for: Planthopper bugs use a fast, cyclic elastic recoil mechanism for effective vibrational communication at small body size
Source: PLoS Biol. 2019 Mar 12;17(3):e3000155. doi: 10.1371/journal.pbio.3000155 (PMC6413918; doi:10.1371/journal.pbio.3000155)
Supplement: S1 Table — Examination of dry mounted specimens using microscopy only allowed documentation of exoskeletal morphology, while musculature was also studied in ethanol-preserved specimens. Use of SR-μCT permitted examination of the exoskeleton, musculature, and innervation of the snapping organ. Illustrations from the literature allowed examination of the external morphology of the vibrational organs of certain delphacids. The abovementioned observation methods allowed us to document the presence of a snapping organ (based on its defining characters) in all examined taxa, with the exception of non-Asiracinae delphacids, the latter having modified snapping organs. BMNH, Natural History Museum, London; DPC, Davranoglou Private Collection; MMBC, Moravian Museum, Brno; OUMNH, Oxford University Museum of Natural History; SR-μCT, synchrotron radiation microcomputed tomography; UG, University of Gdansk (DOCX) [file pbio.3000155.s006.docx]

**S1 Table**

| **Taxon** | **Preservation** | **Observation Method** | **Depository** |
| --- | --- | --- | --- |

| **Acanalonidae** | | |  |  |  |
| --- | --- | --- | --- | --- | --- |
| *A. conica* (Say, 1830) | Dry mounted | | Microscopy | BMNH |  |
| *A. servillei* Spinola, 1839 | Dry mounted | | Microscopy | BMNH |  |
| *A. sublinea* (Walker, 1858) | Dry mounted | | Microscopy | BMNH |  |
| *Acanalonia* sp. 1_Florida_OUMNH-2006-045 | Dry mounted | | Microscopy | OUMNH |  |
| *Acanalonia* sp. 2_Florida_OUMNH-2006-045 | Dry mounted | | Microscopy | OUMNH |  |
| *Acanalonia* sp. Belize_OUMNH-2002-006 | Dry mounted | | Microscopy | OUMNH |  |
| *Acanalonia* sp. Bolivia_OUMNH-2004-005 | Dry mounted | | Microscopy | OUMNH |  |
| *Acanalonia* sp. Argentina_OUMNH-2005-012 | Dry mounted | | Microscopy | OUMNH |  |
| **Achilidae** |  | |  |  |  |
| *Achilus flammeus* Kirby, 1818 | Dry mounted | | Microscopy | BMNH |  |
| *Apateson albomaculatum* Fowler, 1900 | Dry mounted | | Microscopy | MMBC |  |
| *Cixidia skaloula* (Asche, 2015) | ethanol; CPD | | Microscopy; SR-μCT | DPC |  |
| *Errada nebulosa* (Distant, 1914) | Dry mounted | | Microscopy | BMNH |  |
| *Myconus collaris* Melichar, 1904 | Dry mounted | | Microscopy | MMBC |  |
| *Rhotala delineata* Walker, 1857 | Dry mounted | | Microscopy BMNH | |  |
| Achilidae_Burmese_amber | Amber inclusion | | SR-μCT UG | |  |
| **Achilixiidae** | | |  |  |  |
| *Achilixius bakeri* Wilson, 1989 | Dry mounted | | Microscopy | BMNH |  |
| *Bebaoiotes dorsivittata* Fennah, 1947 | Dry mounted | | Microscopy | BMNH |  |
| **Caliscelidae** | | |  |  |  |
| *Bruchomorpha costaricensis* Schmidt, 1927_OUMNH-2009-049 | Ethanol | | Microscopy | OUMNH |  |
| *Caliscelis bonellii* (Latreille, 1807) | ethanol; CPD | | Microscopy; SR-μCT | DPC; BMNH |  |
| *Peltonotellus quadrivittatus* (Fieber, 1876) | Ethanol | | Microscopy | DPC |  |
| **Cixiidae** |  | |  |  |  |
| *Bothriocera westwoodi* (Stal, 1856) | Dry mounted | | Microscopy | MMBC |  |
| Cixiidae sp.1._ Mozambique_OUMNH-2010-098 | ethanol; CPD | | Microscopy; SR-μCT | OUMNH |  |
| *Cixius* sp. | Ethanol | | Microscopy | DPC |  |
| Cixiidae sp.2._Mozambique_OUMNH-2010-098 | ethanol; CPD | | Microscopy; SR-μCT | OUMNH |  |
| *Pentastira* sp. | ethanol; CPD | | Microscopy; SR-μCT | DPC |  |
| *Pentastiridius leporinus* (Linnaeus, 1761) | Dry mounted | | Microscopy | MMBC |  |
| *Tachycixius pilosus* (Olivier, 1791) | Dry mounted | | Microscopy | MMBC |  |
| **Delphacidae** | | |  |  |  |
| *Anakelisia perspicillata* (Boheman, 1845) | image in publication  [1] | | | |  |
| *Asiraca clavicornis* (Fabricius, 1794) | Ethanol | | Microscopy | DPC; BMNH |  |
| Delphacidae sp._Mozambique_OUMNH-2010-098 | ethanol; CPD | | Microscopy; SR-μCT | OUMNH |  |
| *Dicranotropis hamata* (Boheman, 1847) | Ethanol | | Microscopy | DPC; BMNH |  |
| *Elaphodelphax nigropictus* Fennah, 1949 | image in publication  [1] | | | |  |
| Kelisiinae sp._Greece | ethanol; CPD | | Microscopy; SR-μCT | DPC |  |
| *Liburnia britmusei* Asche, 1983 | image in publication  [1] | | | |  |
| *Neopunana saba* Asche, 1983 | image in publication  [1] | | | |  |
| *Stenocranus cf. minutus* (Fabricius, 1787) | ethanol; CPD | | Microscopy; SR-μCT | DPC |  |
| **Derbidae** |  | |  |  |  |
| Zoraidinae sp._Sulawesi_OUMNH-2010-089 | ethanol; CPD | | Microscopy; SR-μCT | OUMNH |  |
| *Derbe pallida* Fabricius, 1803 | Dry mounted | | Microscopy | MMBC |  |
| *Malenia bosnica* (Horvath, 1907) | Dry mounted | | Microscopy | MMBC |  |
| *Phenice signoreti* (Coquerel, 1859) | Dry mounted | | Microscopy | MMBC |  |
| *Proutista moesta* (Westwood, 1851) | Dry mounted | | Microscopy | MMBC |  |
| *Zoraida pterophoroides* (Westwood, 1851) | Dry mounted | | Microscopy | MMBC |  |
| *Z. picta* Distant, 1907 | Dry mounted | | Microscopy | MMBC |  |
| **Dictyopharidae** | | |  |  | |
| *Bursinia genei* (Dufur, 1849) | | ethanol; CPD | Microscopy; SR-μCT | DPC; BMNH | |
| *Callodictya krueperi* (Fieber, 1872) | | Dry mounted | Microscopy | MMBC | |
| *Dictyophara europaea* (Linnaeus, 1767) | | ethanol; CPD | Microscopy; SR-μCT | DPC; BMNH | |
| Orgeriinae sp. | | ethanol; CPD | Microscopy; SR-μCT | DPC | |
| *Parorgerius platypus* (Fieber, 1866) | | Dry mounted | Microscopy | BMNH | |
| *Ranissus edirnaeus* (Dlabola, 1957) | | Dry mounted | Microscopy | MMBC | |
| **Eurybrachidae** | | |  |  | |
| *Aspidonitys trita* Karsch, 1895 | | Dry mounted | Microscopy | BMNH | |
| *Chalia pulchra* (Gray, 1832) | | Dry mounted | Microscopy | OUMNH; BMNH | |
| *Mesonitys fuelleborni* Schmidt, 1908 | | Dry mounted | Microscopy | OUMNH; BMNH | |
| *M. taeniata* (Schmidt, 1906) | | Dry mounted | Microscopy | BMNH | |
| *Platybrachys barbata* (Fabricius, 1775) | | Dry mounted | Microscopy | BMNH | |
| *P. leucostigma* (Walker, 1851) | | Dry mounted | Microscopy | BMNH | |
| **Flatidae** | |  |  |  | |
| Flatidae sp._Mozambique_OUMNH-2010-098 | | ethanol; CPD | Microscopy; SR-μCT | OUMNH | |
| *Hansenia pulverulenta* (Guerin-Meneville, 1844) | | Dry mounted | Microscopy | MMBC | |
| *Ityraea nigrocincta* (Walker, 1858)_ OUMNH-1918-005 | | Dry mounted | Microscopy | OUMNH | |
| *Metcalfa pruinosa* (Say, 1830) | | Dry mounted | Microscopy | MMBC | |
| *Phantia subquadrata* (Herrich-Schaeffer, 1838) | | ethanol; CPD | Microscopy; SR-μCT | DPC; MMBC | |
| *Poeciloptera phalaenoides* (Linnaeus, 1758) | | Dry mounted | Microscopy | MMBC | |
| **Fulgoridae** | | |  |  | |
| *Aphaena aurantia* (Hope, 1840)_ OUMNH-2019-005 | | Dry mounted | Microscopy | OUMNH | |
| *Aracynthus sanguineus* (Olivier, 1791) | | Dry mounted | Microscopy | MMBC | |
| *Cornelia nympha* Stal, 1866 | | Dry mounted | Microscopy | MMBC | |
| *Diareusa imitatrix* Ossiannilsson, 1940_OUMNH-2004-005 | | Dry mounted | Microscopy | OUMNH | |
| *Eddara euchroma* Walker, 1858 | | Dry mounted | Microscopy | MMBC | |
| Fulgoridae sp._Mozambique_OUMNH-2010-098 | | ethanol; CPD | Microscopy; SR-μCT | OUMNH | |
| *Kasserota pupillata* (Stal, 1863) | | Dry mounted | Microscopy | MMBC | |
| *Laternaria phosphorea* (Linnaeus, 1764) | | Dry mounted | Microscopy | MMBC | |
| *Loxocephala decora* (Walker, 1851) | | Dry mounted | Microscopy | MMBC | |
| *Phenax variegata* (Olivier, 1791)_ OUMNH-2019-006 | | Dry mounted | Microscopy | OUMNH | |
| *Phrictus quinquepartitus* Distant, 1883 | | Dry mounted | Microscopy | MMBC | |
| *Pyrops sultana* (Adams & White, 1847)_OUMNH-2011-043 | | Dry mounted | Microscopy | OUMNH | |
| *Saiva transversolineata* (Baker, 1925) Dry mounted Microscopy MMBC | | | | | |
| **Gengidae** | | | | | |
| *Gengis panoblites* Fennah, 1949 Dry mounted Microscopy BMNH | | | | | |
| **Hypochthonellidae** | | | | | |
| *Hypochthonella caeca* China & Fennah, 1952 Dry mounted Microscopy BMNH | | | | | |
| **Issidae** | |  |  |  | |
| *Agalmatium bilobum* (Fieber, 1877) | | ethanol; CPD | Microscopy; SR-μCT | DPC; BMNH | |
| *Atylana herbida* (Walker, 1870) | | Dry mounted | Microscopy | BMNH | |
| *Falcidius apterus* (Fabricius, 1794) | | Dry mounted | Microscopy | MMBC | |
| *Forculus peculiaris* Distant, 1912 | | Dry mounted | Microscopy | BMNH | |
| *Gergithus niger* (Walker, 1857) | | Dry mounted | Microscopy | MMBC | |
| *Glyphotonga acuminata* Schmidt, 1910 | | Dry mounted | Microscopy | BMNH | |
| *Hemisphaerius coccineloides* (Burmeister, 1834) | | Dry mounted | Microscopy | MMBC | |
| Hemispharinae_sp._Mozambique_OUMNH-2010-098 | | ethanol; CPD | Microscopy; SR-μCT | OUMNH | |
| Issidae sp. Sabah_OUMNH-2013-056 | | Ethanol | Microscopy | OUMNH | |
| *Latematium graecicum* (Dlabola, 1982) | | Ethanol | Microscopy | DPC | |
| *Mycterodus pallens* Stal, 1861 | | ethanol; CPD | Microscopy; SR-μCT | DPC | |
| *Oryxana subacuta* (Walker, 1870) | | Dry mounted | Microscopy | BMNH | |
| *Thabenoides opalina* (Distant, 1916) | | Dry mounted | Microscopy | BMNH | |
| *Tonga foliacea* (Stal, 1859) | | Dry mounted | Microscopy | BMNH | |
| *T. guttulata* (Westwood, 1845) | | Dry mounted | Microscopy | BMNH | |
| **Kinnaridae** | | |  |  | |
| *Kinnara ceylonica* (Melichar, 1903) | | Dry mounted | Microscopy | MMBC | |
| *K. flavofasciata* Distant, 1916 | | Dry mounted | Microscopy | BMNH | |
| *Nesomicrixia insularis (Synave, 1958)* | | Dry mounted | Microscopy | BMNH | |
| **Lophopidae** | | |  |  | |
| *Elasmoscelis* sp._Mozambique_OUMNH-2010-098 | | ethanol; CPD | Microscopy; SR-μCT | OUMNH | |
| **Meenoplidae** | | |  |  | |
| *Anigrus bergrothi* (Muir, 1927) | | Dry mounted | Microscopy | BMNH | |
| Meenoplidae sp.Sabah_OUMNH-2013-056 | | Ethanol | Microscopy | OUMNH | |
| Meenoplidae sp._Mozambique_OUMNH-2010-098 | | Ethanol | Microscopy | OUMNH | |
| *Meenoplus albosignatus* Fieber, 1866 | | Dry mounted | Microscopy | MMBC | |
| *Phaconeura fletcheri* Kirkaldy, 1908 | | Dry mounted | Microscopy | BMNH | |
| **Nogodinidae** | | |  |  | |
| *Biolleyana fenestra* (Gerstaecker, 1895) | | Dry mounted | Microscopy | MMBC | |
| *Indogaetulia nigrovenosa* (Melichar, 1898) | | Dry mounted | Microscopy | BMNH | |
| *Mindura simiana* Distant, 1910 | | Dry mounted | Microscopy | BMNH | |
| *Monteira cornicula* Melichar, 1906 | | Dry mounted | Microscopy | BMNH | |
| Nogodinidae sp._Mozambique_OUMNH-2010-098 | | ethanol; CPD | Microscopy; SR-μCT | OUMNH | |
| Nogodinidae sp. Honduras | | Ethanol | Microscopy | DPC | |
| *Psiadiicola brevipennis* Fennah, 1978 | | Dry mounted | Microscopy | BMNH | |
| *Sassula concolor* (Walker, 1870) | | Dry mounted | Microscopy | BMNH | |
| *Varcia greeni* (Kirby, 1891) | | Dry mounted | Microscopy | MMBC | |
| *V. pyramidalis* Melichar, 1898 | | Dry mounted | Microscopy | MMBC | |
| **Ricaniidae** | | |  |  | |
| *Pocharista conradti* (Schmidt, 1906) | | Dry mounted | Microscopy | MMBC | |
| *Pochazia flavocostata* Melichar, 1898 | | Dry mounted | Microscopy | MMBC | |
| *Ricania discoptera* Stal, 1865 | | Dry mounted | Microscopy | MMBC | |
| *R. trimaculata* Guerin-Meneville, 1838 | | Dry mounted | Microscopy | MMBC | |
| *Ricanoptera mellerborgi* (Lallemand, 1854) | | Dry mounted | Microscopy | MMBC | |
| **Tettigometridae** | | |  |  | |
| *Euphyonarthex* sp._Cameroon_OUMNH-2019-004 | | Dry mounted | Microscopy | OUMNH | |
| *Tettigometra atra* Hagenbach, 1825 | | Dry mounted | Microscopy | MMBC | |
| *T. impressifrons* Mulsant & Rey, 1855 | | Ethanol | Microscopy | DPC | |
| *T. laeta* Herrich-Schaeffer, 1835 | | ethanol; CPD | Microscopy; SR-μCT | DPC | |
| *T. leucophaea* (Preyssler, 1792) | | Dry mounted | Microscopy | MMBC | |
| **Tropiduchidae** | | |  |  | |
| *Cixiopsis punctatus* Matsumura, 1900 | | Dry mounted | Microscopy | BMNH | |
| *Eilithyia insularis* Distant, 1912 | | Dry mounted | Microscopy | BMNH | |
| *Epora montana* Distant, 1912 | | Dry mounted | Microscopy | BMNH | |
| *Eporiella ceylonica* Melichar, 1914 | | Dry mounted | Microscopy | BMNH | |
| *Isporisa apicalis* Walker, 1857 | | Dry mounted | Microscopy | BMNH | |
| *Leusaba* *marginalis* Walker, 1857 | | Dry mounted | Microscopy | BMNH | |
| *Ommatissus binotatus* Fieber, 1876 | | Dry mounted | Microscopy | MMBC | |
| *Padanda denti* Muir, 1934 | | Dry mounted | Microscopy | BMNH | |
| *Paricana dilatipennis* Walker, 1857 | | Dry mounted | Microscopy | BMNH | |
| *Pseudoclardea leguati* (Muir, 1925) | | Dry mounted | Microscopy | BMNH | |
| *Stacota breviceps* (Walker, 1858) | | Dry mounted | Microscopy | BMNH | |
| *Trypetimorpha fenestrata* Costa, 1862 | | Dry mounted | Microscopy | MMBC | |
| Tropiduchidae sp.Sabah_OUMNH-2013-056 | | Ethanol | Microscopy | OUMNH | |
| *Vanua respicienda* (Walker, 1858) | | Dry mounted | Microscopy | BMNH | |
| *Varma tridens* Distant, 1906 | | Dry mounted | Microscopy | BMNH | |

**Reference**

1. Asche M. Zur phylogenie der Delphacidae Leach, 1815 (Homoptera Cicadina Fulgoromorpha). Marburg Entomol Publ. 1985; 2: 1-398.
