# Supplementary material for: Planthopper bugs use a fast, cyclic elastic recoil mechanism for effective vibrational communication at small body size
Source: PLoS Biol. 2019 Mar 12;17(3):e3000155. doi: 10.1371/journal.pbio.3000155 (PMC6413918; doi:10.1371/journal.pbio.3000155)
Supplement: S2 Table — Function of muscles was inferred by high-speed videography, power calculations of laser Doppler vibrometry recordings of A. bilobum, and artificial contraction of the respective muscles using a pair of forceps in ethanol-preserved specimens. SR-μCT, synchrotron radiation microcomputed tomography (DOCX) [file pbio.3000155.s007.docx]

**S2 Table**

| **Muscle** | **Origin** | **Insertion** | **Inferred function** |
| --- | --- | --- | --- |

| **Idlm1** | Apodeme on metaphragma | Apodeme of tergum two | Raises the abdomen; acts a spring, storing and releasing energy during loading phase |
| --- | --- | --- | --- |
| **Idlm2** | Strip of tergum one | Tergum two | Assists in closure of Y-lobe arms |
| **Idvm** | Postcoxale | Base of Y-lobe, immediately above connector, beneath vertical list | Pulls Y-lobe downward |
| **IIedvm1** | Apical third of stnIIa | Proximal portion of spine-like apodeme of tergum two | Moves spine-like apodeme (and as a result base of Y-lobe as well) downward, causing closure of Y-lobe arms during loading phase |
| **IIedvm2** | Apex of stnIIa | Distal portion of spine-like apodeme of tergum two | Moves spine-like apodeme (and as a result base of Y-lobe as well) downward, causing closure of Y-lobe arms during loading phase |
| **IIidvm1** | Small apodeme on median region of stnnIIa | Membrane immediately anterior to base of Y-lobe | Unknown |
| **IIidvm2** | Apical third of stnIIa, slightly lower than IIedvm2 | Connector arm of tergum two | Assists in closure of Y-lobe arms |
| **IIisdvm** | Apex of stnIIb | Anterior margin of tergum three | Unknown |
| **IIIvlm2** | Metafurca | Antecosta of segment 1 | Moves abdomen downward |
| **Ivlm** | Base of postcoxale | Antecosta of segment 1 | Assists in moving abdomen downward |
| **IIvlm1** | Sternum IIa | Sternum IIb | Contraction of Sternum IIa-b |
