# Supplementary material for: Planthopper bugs use a fast, cyclic elastic recoil mechanism for effective vibrational communication at small body size
Source: PLoS Biol. 2019 Mar 12;17(3):e3000155. doi: 10.1371/journal.pbio.3000155 (PMC6413918; doi:10.1371/journal.pbio.3000155)
Supplement: S3 Table — Inferences of homology and segmental identity were based on muscle innervation and location from SR-μCT and ethanol-preserved specimens. En-dash (–) denotes that the character is either absent or not reported by the study in question. SR-μCT, synchrotron radiation microcomputed tomography (DOCX) [file pbio.3000155.s008.docx]

**S3 Table**

| **Taxon:** | ***Dicranotropis* *hamata* (Boheman, 1847)** | ***Nilaparvata* *lugens* (Stal, 1854)** | ***Stenocranus* *fuscovittatus***  **(Stal, 1858)** |
| --- | --- | --- | --- |

| **Muscle** | |  |  | |  |  |
| --- | --- | --- | --- | --- | --- | --- |
| Idlm1 | | Iadlm1 | DLM1 | | Iadlm1 |  |
| Idlm2 | | Iadlm2 | DLM2 | | - |  |
| IIIvlm2 | | IIIvlm | VLM1 | | - |  |
| Ivlm1 | | - | - | | - |  |
| IIvlm1 | | Iavlm1 | VLM4 | | - |  |
| IIvlm2 | | Iavlm2 | VLM2 | | Iavlm2 |  |
| IIvlm3 | | - | VLM3 | | - |  |
| Idvm1 | | - | - | | - |  |
| IIidvm1 | | Iadvm1 | DVM1 | | Iadvm1 |  |
| IIidvm2 | | - | - | | - |  |
| IIedvm1 | Iadvm2 | | DVM2 | Iadvm2 | | |
| IIedvm2 | Iadvm3 | | DVM3 | - | | |
| IIisdvm | - | | ISM1 | - | | |
| **Author:** | [1] | | [2] | [3] | | |

**References**

1. Ossiannilsson F. Insect drummers. A study on the morphology and function of the sound-producing organ of swedish Homoptera Auchenorrhyncha. Op Ent Suppl 10. 1949; 3: 1-146.

2. Mitomi M, Ichikawa T & Okamoto H. Morphology of the vibration-producing organ in adult rice brown planthopper, *Nilaparvata lugens* (Stal) (Homoptera: Delphacidae). Appl Entomol Zool. 1984; 19: 407-417.

3. Asche M. Zur phylogenie der Delphacidae Leach, 1815 (Homoptera Cicadina Fulgoromorpha). Marburg Entomol Publ. 1985; 2: 1-398.
