# Supplementary material for: Planthopper bugs use a fast, cyclic elastic recoil mechanism for effective vibrational communication at small body size
Source: PLoS Biol. 2019 Mar 12;17(3):e3000155. doi: 10.1371/journal.pbio.3000155 (PMC6413918; doi:10.1371/journal.pbio.3000155)
Supplement: S4 Table — Characters of delphacids largely based on the meta-analysis of Asche, 1990 [34] and our own observations of ethanol-preserved and SR-μCT specimens. Order of character states does not imply evolutionary sequence. SR-μCT, synchrotron radiation microcomputed tomography (DOCX) [file pbio.3000155.s009.docx]

**S4 Table**

| **Character number** | **Character state** |  |
| --- | --- | --- |
| **Non-Asiracinae Delphacidae** | |  |
| 1 | Snapping organ sexually dimorphic | |
| 2 | Ventrocaudal enlargement of Idlm1-bearing apodemes and  migration to median region of metapostnotum | |
| 3 | Enlargement of dorsoventral musculature and IIvlm2 | |
| 4 | Fusion and modification of Y-lobe | |
| 5 | Enlarged sternal apodemes for IIvlm2 | |
| 6 | Detachment of spiracle lobe from tergum two and partial fusion to Y-lobe | |
| 7 | Development of central plate on tergum two | |
| **Derbidae (non-Cedusinae)** |  | |
| 8 | Y-lobe externally obscure, extremely flat, covered by ridge and membranes | |
| 9 | Metathoracic wing stridulatory device | |
