# Supplementary material for: Planthopper bugs use a fast, cyclic elastic recoil mechanism for effective vibrational communication at small body size
Source: PLoS Biol. 2019 Mar 12;17(3):e3000155. doi: 10.1371/journal.pbio.3000155 (PMC6413918; doi:10.1371/journal.pbio.3000155)
Supplement: S1 Text — (DOCX) [file pbio.3000155.s013.docx]

S1 Text

Extended description of the snapping organ of *Agalmatium bilobum* (Issidae)

1. Exoskeleton

The morphology of the first and second abdominal segments is complex. A narrow, sclerotized strip fused to the metapostnotum and metepimeron represents the anterior-most part of the first abdominal tergum (Figs 1B, 2A, yellow). This strip bears the first abdominal spiracle, a pair of arm-like ridges (Fig 1B, rg), and is ventrally continuous with the postcoxale (formed by the first abdominal segment; Fig 2A, yellow). The opposing ridges fuse dorsally (Fig 1B) on the anterior arm of a modified, Y-shaped lobe of tergum one (Figs 1B, rg, 2A, brown). A distinctive seta is present in the dorsal portion of each ridge (not shown). The sclerotized metapostnotal strip and the Y-lobe are separated by a folded membrane (Fig 1B). The anterior arm of the Y-lobe is continuous dorsally, while its posterior arm fuses to tergum two (Fig 1B, tg 2) and dorsomedially terminates adjacent to a pair of apodemes of segment two (S1 Fig, Fig 2B, apo), which serve as attachments for the hypertrophied Idlm1 muscles (Fig 2B, apo). The two arms of the Y-lobe are separated by a membrane containing resilin, separated from each other at the midline by a segment of cuticle (S1 Fig). Tergum two is subdivided into a proximal sclerotized portion (Fig 1B, green) and a distal membranous, cushion-like pad. The lateral margin of the sclerotized portion of tergum two bears posteriorly a pair of connector arms, which link it to tergum three. The spiracle of the second abdominal segment is fused to tergum two, forming the proximal portion of the segments’ lateral margin (Fig 1B). The lateral margin of tergum two is crescent-shaped, distinctly separating the fused lobe of the second abdominal spiracle and the connector arms (Figs 1B, 2A). Immediately beneath spiracle two, a small orifice forms internally a downward-facing spine (Fig 2C, sp). A connective from the base of the Y-lobe fuses to this spine-like apodeme, linking the two segments functionally and morphologically (Figs 1B, cn, 2A).

Both segments are characterized by subdivided sternites one and two (Fig 2A). The anterior and posterior margins of each sternite are sclerotized, while their median region is divided by a broad membrane. The first subdivision of sternum one (stnIa) is fused to the metafurca and forms the postcoxale (Fig 2A, yellow), while the second (stnIb) is represented by a short sclerotized flap, immediately anterior to sternum two. stnIIa and stnIIb are interrupted by a membrane, like the previous segment and are also strip-like. A small apodeme protrudes from the median region of stnIIa (not shown), while stnIIb is closely associated with segment three both dorsally and ventrally. Both subdivisions of the second segment terminate into a spatulate apex (Fig 2A).

2. Musculature and nervous system

The segmental affinities of the dorsoventral muscles and ventral sclerites operating the snapping organ and the tymbal-like organs of Cicadomorpha have been contentious, with some authors assigning them to the first abdominal segment [1, 2] and others to the second [3, 4]. Based on innervation as observed by SR-µCT (S2 Fig), the primary dorsoventral muscles attaching on the internal spine of the second abdominal tergum (Fig 2A, C) are innervated by the second abdominal nerve (S2 Fig, n.ab. 2) and belong to the second abdominal segment. The two main dorsal longitudinal muscles – the hypertrophied Idlm1 and the smaller Idlm2 (Fig 2A), are innervated by the first abdominal nerve (S2 Fig, n.ab. 1) and belong to the corresponding segment. A summary of the origins and insertions of all muscles involved in the snapping organ, together with their inferred functions, is provided in S2 Table.

The snapping organ in other planthopper families

The exoskeletal components defining the snapping organ of our model species, *A. bilobum*, i.e. the characteristic arrangement of the ridge, Y-lobe and connector, were found in all 21 examined planthopper families (S1 Table). Although the overall morphology and arrangement of the snapping organ exoskeleton is remarkably constant, there is some variation in the general shape of its components (Figs 2, 3), with the main variants described below:

1) The **ridge** in all examined taxa acts as a bridge between the first abdominal segment strip (fused to the metapostnotum) and the anterior Y-lobe arm. However, it may cover only a small portion of the anterior Y-lobe arm (e.g. in some Cixiidae and Asiracinae, Fig 3B, C), or completely envelop it and fuse with the ridge on the other side of the organ (e.g. Caliscelidae, Fig 2E, tettigometrids). The distinct seta on each ridge is invariably present in all examined planthoppers.

2) The **Y-lobe** is typically Y-shaped, with anterior and posterior arms, connecting to the ridge and tergite two respectively. The Y-lobe base may vary significantly in shape, ranging from short and robust (e.g. cixiids, Fig 3B), to elongate (most planthoppers, Figs 1B, 3C, D). In addition, the Y-lobe may be entirely transparent in small, soft bodied forms (e.g. many Meenoplidae, Kinnaridae and Issidae). A characteristic shared by all examined Dictyopharidae and Fulgoridae, is that the posterior arm of the Y-lobe and the apodemes of Idlm1 on tergite two are not visible externally.

3) The **connector** always links the base of the Y-lobe to the base of tergite two. The connector may be entirely membranous in some taxa (e.g. some Issidae), or considerably sclerotized (e.g. tettigometrids). Its shape may vary from very narrow (e.g. Issidae, Figs 1B, 2) to quite broad (e.g. Flatidae, Nogodinidae, Ricaniidae).

4) **Tergite two** is generally quite uniform. The median incision between the apodemes for Idlm1 may be shallow (many Issidae, Flatidae, Caliscelidae, Lophopidae etc.), extending to more than half of the tergite length (e.g. Fig 3B, C, D), or completely absent (many Dictyopharidae and Fulgoridae). Tergite two may be distinctly subdivided into an anterior sclerotized part and a posterior membranous region (Fig 1). The membranous region may be very small (Fig 3B, C), interrupted by a median sclerotized region (Fig 3D), or entirely absent (Fig 3E). The anterior sclerotized region may frequently be raised, strongly convex (e.g. most ricaniids, flatids, nogodinids), slightly less raised (e.g. most eurybrachids) or almost entirely flat (e.g. most Issidae, Fig 1B).

We have not observed sexual dimorphism in the morphology of the snapping organ in any of the examined species (S1 Table).

All the above exoskeletal variations can be found in various combinations throughout planthoppers, which suggests that these variations have arisen many times independently. The musculature defining the snapping organ is even more uniform in 11 of the 12 families examined using SR-μCT (S1 Table), consistent with a single origin at the base of fulgoromorphan phylogeny (Fig 3). The examined non-cedusine derbids are an exception, in which the Y-lobe is externally obscure (S3 Table), and is supplemented by a tentative wing stridulatory mechanism.

The subtle variations in the external morphology of the snapping organ beyond the given defining characters between planthopper families are likely to be of significant systematic and taxonomic importance, and more detailed studies are necessary. The modified organs of delphacids are species-specific and already used in both taxonomic and systematic studies [3, 5]; we expect that the same holds true for the remaining planthoppers.

Overall, the defining exoskeletal elements and muscles of the snapping organ are constant across the examined planthopper taxa (with the exception of some delphacids, Fig 2; see below). The above suggest that the vibrational mechanism is also conserved, which is supported by the overall uniformity in signal structure of most planthoppers [6, 7]. Given that the earliest definitive planthoppers originated in the Jurassic (260 million years ago) [8, 9], the snapping organ represents a remarkably conserved bauplan. However, it is possible that additional, as yet undiscovered components or vibration generation mechanisms may also supplement the calls of planthoppers possessing a snapping organ.

The delphacid “drumming organ”

The vibrational organ (also known as drumming organ) of non-asiracine delphacids has long been thought to represent the mechanism of vibration generation of planthoppers as a whole [3, 5, 6, 10]. In contrast, we suggest that the drumming organ of delphacids is a modified snapping organ, sharing the same bauplan: A series of cumulative exoskeletal and muscle transformations (S3 Table) [5] in non-Asiracinae Delphacidae has led to the development of a very specialized structure, which presumably functions differently from the snapping organs of other planthoppers. Although the defining characteristics of the snapping organ are present, the vibrational organs of non-Asiracinae have undergone such drastic reorganization and addition of novel components, such as the central plate (S3A Fig), which makes it unlikely that they function in the same way as snapping organs. Given that the homologies of the vibrational organs of planthoppers and non-Asiracinae have never been elucidated, we briefly describe below our interpretations of the exoskeleton and musculature of the delphacid drumming organ.

We describe the exoskeletal components of the drumming organ in a generalized delphacid – *Stenocranus* *minutus*, which would be applicable for most non-Asiracinae delphacids. The metapostonotal apodemes are centrally located, and strongly expanded ventrally (S3 Table). Each ridge is greatly expanded, spatulate in shape (S3A, B, C Fig, yellow). The distinctive seta found on the ridge of other planthoppers is present, but located towards the base of the ridge. The two opposing ridges are separated by a transparent cuticular bridge of uncertain origin (S3A, B, C Fig, pink) – it may represent an unmodified section of tergite one, or an expansion of the anterior Y-lobe arm. The entire Y-lobe is strongly reduced to a small rounded lobe, fused to the posterior lateral margin of each ridge (S3A, B, C Fig, lb, brown). The Y-lobe rudiment bears a transverse list, of uncertain function (S3C Fig, lt). Due to the rudimentary and modified nature of the Y-lobe in non-Asiracinae delphacids, we cannot ascertain whether the posterior Y-lobe arm is either strongly reduced or completely absent. The entire median region of tergite two has been transformed into a central plate (S3A, B Fig, cp) which internally supports large apodemes for the greatly enlarged Idlm1 (S3B Fig).

We examined the musculature of the drumming organ of delphacids by using SR-μCT in *S. minutus* and compared them with illustrations from previous morphological studies [3-5] and SR-μCT scans of planthoppers from the other 11 planthopper families (S1 Table). We observed that the drumming organs (i.e. the modified snapping organs of non-Asiracinae delphacids) use the same musculature with the rest of Fulgoromorpha (S3D Fig, S3, S4 Tables), although their relative size (and probably function as well) is different. In non-Asiracinae delphacids, exemplified here by *S. minutus*, there is a general trend for enlargement of the dorsoventral and longitudinal musculature of the first two abdominal segments: muscles that are small (IIIvlm3, IIvlm2, IIedvm1) or thread-like (e.g. IIidvm1) in most Fulgoromorpha are greatly enlarged in non-Asiracinae delphacids (S3D Fig). Muscle Idlm1, which is already hypertrophied in all examined planthoppers, is even larger in non-Asiracinae delphacids [3, 4]. In addition, the downward expansion of the metapostnotal apodemes, have provided this muscle with a near-dorsoventral position, with possible functional implications. We found muscle Idvm in *S. minutus*, which has not been reported previously from delphacids (S3D Fig, S4 Table), while we could not locate IIidvm2. There may be variation in the size of the different dorsoventral muscles in different delphacid taxa; in *S. minutus*, II edvm1 is the largest dorsoventral muscle, whereas in *Nilaparvata lugens*, IIidvm1 is the largest [4].

**References**

1. Snodgrass RE. Morphology of the insect abdomen. 1. General structure of the abdomen and its appendages. Smith Misc Coll. 1931; 85: 1-128.

2. Kramer S. The morphology and phylogeny of auchenorrhynchous Homoptera (Insecta). Urbana-Champagne: University of Illinois Press; 1950.

3. Ossiannilsson F. Insect drummers. A study on the morphology and function of the sound-producing organ of swedish Homoptera Auchenorrhyncha. Op Ent Suppl 10. 1949; 3: 1-146.

4. Mitomi M, Ichikawa T & Okamoto H. Morphology of the vibration-producing organ in adult rice brown planthopper, Nilaparvata lugens (Stal) (Homoptera: Delphacidae). Appl Entomol Zool. 1984; 19: 407-417.

5. Asche M. Vizcayinae, a new subfamily of Delphacidae with revision of Vizcayia Muir (Homoptera: Fulgoroidea) - a signficiant phylogenetic link. Bishop Mus Occ Papers. 1990; 30: 154-187.

6. Tishechkin DY. On the similarity of temporal pattern of vibrational calling signals in different species of Fulgoroidea (Homoptera: Auchenorrhyncha). Russ Entomol J. 2008; 17: 349-357.

7. Tishechkin DY. On the similarity of temporal pattern of vibrational calling signals in different species of Fulgoroidea (Homoptera: Auchenorrhyncha). Russ Entomol J. 2008; 17: 349-357.

8. Song N, & Liang AP. A Preliminary Molecular Phylogeny of Planthoppers (Hemiptera: Fulgoroidea) Based on Nuclear and Mitochondrial DNA Sequences. PLoS ONE. 2013; 8: e58400.

9. Urban JM & Cryan JR. Evolution of the planthoppers (Insecta: Hemiptera: Fulgoroidea). Mol Phyl Evol. 2007; 42: 556-572.

10. Wessel A, Mühlethaler R, Hartung V, Kustor V & Gogala M. The tymbal: evolution of a complex vibration-producing organ in the Tymbalia (Hemiptera excl. Sternorrhyncha). In: Cocroft RB, Gogala M, Hill PSM & Wessel A, editors. Animal signals and communication: studying vibrational communication. Berlin: Springer; 2014. pp. 395-444.
