# Supplementary material for: Correction: Planthopper bugs use a fast, cyclic elastic recoil mechanism for effective vibrational communication at small body size
Source: PLoS Biol. 2020 Dec 9;18(12):e3001047. doi: 10.1371/journal.pbio.3001047 (PMC7725284; doi:10.1371/journal.pbio.3001047)
Supplement: S1 Methods — (DOCX) [file pbio.3001047.s002.docx]

S1 Methods

Insects

Larvae of *Agalmatium bilobum* were collected on *Asphodelus fistulosus*, whereas adults were collected from a variety of other plants. Larvae were reared with their potted host plant or daffodils (*Narcissus pseudonarcissus*). Many adults exhibited wing deformities from improper moulting, indicating that humidity levels were not optimal. Such individuals were excluded from our study. Adults were separated from each other as soon as they moulted and placed in unisex meshed cages.

Morphological analysis

To investigate the presence of resilin in the snapping organ of *A. bilobum* (S1 Fig), the thorax and the first three abdominal segments were dissected from five chilled individuals (0 ^o^C for 5 minutes). The dissected snapping organs were immediately placed in excavated microscope slides and viewed through a Leica DM2000 LED under ultraviolet (UV) illumination at 365 nm, using a general blue (465/20)/green (530/30)/red (640/40) bandpass filter and a MC120 HD camera. Images captured at the same focal planes under UV and visible light were superimposed using Photoshop CS6.

Power calculations

The mid-abdomen recordings taken orthogonal to the thorax were analysed to extract the co-ordinates of the maximum and minimum velocities of the loading and unloading steps and the last x axis crossing before the absolute maximum peak (S1 Data). The peak kinetic energy of the motion was calculated from the speed of the measured dorsoventral translation of the abdominal mass, multiplying its square by 0.5 times the abdomen mass (10.33 mg). For mass estimation, the abdomen of a single female adult *A. bilobum* was excised and measured in a [Sartorius CPA225D](https://balance.balances.com/scales/854) semi-micro balance.

To calculate the power, the energy was divided by the time taken to reach peak velocity from rest. To calculate the specific power and energy, muscle mass was required. We assumed a muscle density of 1060 kg m^-3^, and estimated muscle volume directly from the micro-CT data by counting the number of (1.625 µm)^3^ voxels occupied by each muscle, having segmented them using Amira 6.1 (ThermoFisher Scientific, MA, USA). We calculated the maximum muscle mass, which was the pair of DLMs (Idlm1, Idlm2) added to the smaller paired DVM muscles (IIedvm1, IIedvm2, Fig 2, S1 Data). Specific power was then calculated as the power divided by paired muscle mass from all four muscles. Specific energy was the energy divided by the paired DLM muscle mass for both DLMs.

Mathematical model

A simple mathematical model was developed to investigate the vibration of the snapping organ during the unloading phase. The mathematical model consists of a massless rigid bar connected to a fixed surface at one end and to another massless rigid bar at the other, which had a lumped mass at its other end (Fig 5A, S4 Fig). The lumped mass represented the abdomen, the 10.33 mg mass of which was obtained through measurement, and the two rigid bars represented the moving parts of the snapping organ (ridge and anterior arm of the Y-lobe). Two torsion springs with stiffness *k_3_* and *k_4_* account for the relative movements of the rigid bars, and two linear springs with stiffness *k_1_* and *k_2_* represent the contribution of the muscles and cuticle with resilin to the dorsoventral and longitudinal vibration of the abdomen. Damping effects were included by using a viscous damping model, using two viscous dashpots with damping coefficients *λ_1_* and *λ_2_*, as shown in S4 Fig). Only planar motion was considered, and only the unloading phase was modelled to test whether the linear model was able to capture the motion measured using laser vibrometry during that phase.

The angles between the thorax and ridge, and the ridge and Y-lobe during the relaxed (unloaded) phase are respectively, $\tilde{\theta}$*_1_* = 15° and $\tilde{\theta}$*_2_* = 13.8° and they are taken as the nominal position. When loaded these angles take the values of $\hat{\theta}_{1}$= 30° and $\hat{\theta}_{2}$= 2.4°, respectively. The angles in the unloaded and loaded phases of the vibration generation mechanism were measured by post-processing the high-speed video recordings. The lengths of the ridge (228 µm) and Y-lobe (257 µm), as well as the length and angle of the muscles (used to calculate *k_1_* and *k_2_*) were measured from micro-CT data. ImageJ (NIH) was used to evaluate angles and length of images. An approximation of the spring stiffnesses *k_3_* (0.31 N µm rad^-1^) and *k_4_* (0.62 N µm rad^-1^) were calculated from an experiment where a known weight was hung from the anterior arm of the Y-lobe at the midline. Each rotation spring was calculated by measuring the angle $\varphi$ of rotation of the arm from the unloaded and loaded configurations, and the distance between the applied weight and the center of rotation. Then, using the well-known relationship between the torque generated by the weight and the rotation, *T*= *k* $\varphi$, the rotational stiffness *k* was determined. Photographs of the angles of the ridge and Y-lobe before and after mass was applied were taken using a Leica M165c microscope equipped with a Leica DFC490 camera. The remaining parameters, *k_1_*, *k_2_*, *λ_1_* and *λ_2_* (0.01 N µm^-1^, 6.00 N µm^-1^, 0.000012 N s µm^-1^ and 0.00055 N s µm^-1^), were chosen to best match the model motion in the dorso-ventral plane with the measured motion, in order to capture the amplitude and timing of the first two peaks (Fig 5).

$\tilde{\theta}_{1},\tilde{\theta}_{2}$describe the nominal position, that is the position about which we want to study the vibration. The parameters $\theta_{1},\theta_{2}$ are the generalised coordinates that describe the rotation of the rigid bars about the nominal position. These are the minimum number of parameters needed to describe the motion of the mass in the x and y direction. Indicating with $L_{OB},L_{BC}$ the length of the bars OB and BC, respectively, the position of the mass in the x and y direction (with respect to the origin 0) can be expressed as:

$x_{c}=L_{OB}\sin\left(\tilde{\theta}_{1}-\theta_{1}\right)+L_{BC}\sin\left(\tilde{\theta}_{2}+\theta_{2}\right)$ (1)

$y_{c}=L_{OB}\cos\left(\tilde{\theta}_{1}-\theta_{1}\right)-L_{BC}\cos\left(\tilde{\theta}_{2}+\theta_{2}\right)$ (2)

By differentiation with respect to time, the velocity in the *x* and *y* direction are obtained:

$v_{xc}=-L_{OB}\cos\left(\tilde{\theta}_{1}-\theta_{1}\right)\theta'_{1}+L_{BC}\cos\left(\tilde{\theta}_{2}+\theta_{2}\right)\theta'_{2}$ (3)

$v_{yc}=L_{OB}\sin\left(\tilde{\theta}_{1}-\theta_{1}\right)\theta'_{1}+L_{BC}\sin\left(\tilde{\theta}_{2}+\theta_{2}\right)\theta'_{2}$ (4)

The equations describing the motion of this system with respect to the nominal position can be determined by using the well-known Lagrangian approach [1]. This approach requires the evaluation of the kinetic energy, potential energy and dissipation function of the system. The kinetic energy of the system is given by the kinetic energy of the mass in the *x* and *y* direction. This can be expressed as:

$T=\frac{1}{2}mv_{xc}^{2}+\frac{1}{2}mv_{yc}^{2}$ (5)

The potential energy of the system is given by the potential energy associated with the two translation springs of stiffness *k_1_* and *k_2_*, with the torsion springs *k_3_* and *k_4_*, and with the potential energy associated with the gravity loading, so that:

$V=\frac{1}{2}k_{1}x_{c}^{2}+\frac{1}{2}k_{2}y_{c}^{2}+\frac{1}{2}k_{3}\left(\tilde{\theta}_{1}-\theta_{1}\right)^{2}+\frac{1}{2}k_{4}\left(\tilde{\theta}_{1}-\theta_{1}+\tilde{\theta}_{2}+\theta_{2}\right)^{2}+mgy_{c}$ (6)

The dissipation function is given by the dissipation associated with the translation dampers with damping coefficients *λ_1_* and *λ_2_*:

$D=\frac{1}{2}\lambda_{1}v_{xc}^{2}+\frac{1}{2}\lambda_{2}v_{yc}^{2}$ (7)

The governing equations of motion in terms for the free vibration analysis (no external force is acting on the system, only initial conditions are applied corresponding to the loaded angle configuration) can now be determined by writing the Lagrange’s equations [1] of this two-degrees of freedom system:

(8)

(9)

Equations 8 and 9 represent a system of two non-linear equations. We can now linearize the system of equations with respect to the nominal position $\tilde{\theta}_{1}$ and *_2_* $\tilde{\theta}_{2}$to describe the small amplitude oscillations about these positions, so that:

$\sin\left(\tilde{\theta}_{1}-\theta_{1}\right)=\sin\left(\tilde{\theta}_{1}\right)-\theta_{1}\cos\left(\tilde{\theta}_{1}\right)$ (10)

$\cos\left(\tilde{\theta}_{1}-\theta_{1}\right)=\cos\left(\tilde{\theta}_{1}\right)+\theta_{1}\sin\left(\tilde{\theta}_{1}\right)$ (11)

$\sin\left(\tilde{\theta}_{2}+\theta_{2}\right)=\sin\left(\tilde{\theta}_{2}\right)+\theta_{2}\cos\left(\tilde{\theta}_{2}\right)$ (12)

$\cos\left(\tilde{\theta}_{2}+\theta_{2}\right)=\cos\left(\tilde{\theta}_{2}\right)-\theta_{2}\sin\left(\tilde{\theta}_{2}\right)$ (13)

Substituting these expressions and retaining only the linear terms, the following system of equations assembled in matrix form is obtained:

(14)

$\begin{matrix} K_{11}=k_{3}+k_{4}+ \\ \begin{matrix} & \end{matrix}+L_{OB}\left\{\left(k_{1}-k_{2}\right)L_{OB}\cos\left(2\tilde{\theta}_{1}\right)+\cos\left(\tilde{\theta}_{1}\right)\left[-gm+k_{2}L_{BC}\cos\left(\tilde{\theta}_{2}\right)\right]-k_{1}L_{BC}\sin\left(\tilde{\theta}_{1}\right)\sin\left(\tilde{\theta}_{2}\right)\right\} \end{matrix}$ (15)

$K_{12}=-k_{4}+L_{OB}L_{BC}\left(k_{2}\sin\left(\tilde{\theta}_{1}\right)\sin\left(\tilde{\theta}_{2}\right)-k_{1}\cos\left(\tilde{\theta}_{1}\right)\cos\left(\tilde{\theta}_{2}\right)\right)$ (16)

$K_{21}=-k_{4}+L_{OB}L_{BC}\left(k_{2}\sin\left(\tilde{\theta}_{1}\right)\sin\left(\tilde{\theta}_{2}\right)-k_{1}\cos\left(\tilde{\theta}_{1}\right)\cos\left(\tilde{\theta}_{2}\right)\right)$ (17)

$\begin{matrix} K_{22}=k_{4}+ \\ \begin{matrix} & \end{matrix}+L_{BC}\left\{\left(k_{1}-k_{2}\right)L_{BC}\cos\left(2\tilde{\theta}_{2}\right)+\cos\left(\tilde{\theta}_{2}\right)\left[gm+k_{2}L_{OB}\cos\left(\tilde{\theta}_{1}\right)\right]-k_{1}L_{OB}\sin\left(\tilde{\theta}_{1}\right)\sin\left(\tilde{\theta}_{2}\right)\right\} \end{matrix}$ (18)

$\begin{matrix} Q_{1}=-\left(k_{3}+k_{4}\right)\tilde{\theta}_{1}-k_{4}\tilde{\theta}_{2}+gmL_{OB}\sin\left(\tilde{\theta}_{1}\right)+\left(k_{2}-k_{1}\right)L_{OB}^{2}\cos\left(\tilde{\theta}_{1}\right)\sin\left(\tilde{\theta}_{1}\right)+ \\ \begin{matrix} & \end{matrix}-L_{OB}L_{BC}\left(k_{1}\cos\left(\tilde{\theta}_{1}\right)\sin\left(\tilde{\theta}_{2}\right)+k_{2}\cos\left(\tilde{\theta}_{2}\right)\sin\left(\tilde{\theta}_{1}\right)\right) \end{matrix}$ (19)

$\begin{matrix} Q_{2}=\left(\tilde{\theta}_{1}+\tilde{\theta}_{2}\right)k_{4}+gmL_{BC}\sin\left(\tilde{\theta}_{2}\right)-\left(k_{2}-k_{1}\right)L_{BC}^{2}\cos\left(\tilde{\theta}_{2}\right)\sin\left(\tilde{\theta}_{2}\right)+ \\ \begin{matrix} & \end{matrix}+L_{OB}L_{BC}\left(k_{1}\cos\left(\tilde{\theta}_{2}\right)\sin\left(\tilde{\theta}_{1}\right)+k_{2}\cos\left(\tilde{\theta}_{1}\right)\sin\left(\tilde{\theta}_{2}\right)\right) \end{matrix}$ (20)

The system of two linear equations (Eq. (14)) can now be solved to determine the angular velocities and their derivatives with respect to time when the initial loaded conditions in terms of $\hat{\theta}_{1}$ and $\hat{\theta}_{2}$are assigned. From that, using Eq. (1-4), it is then possible to determine the mass position and velocity in the *x* and *y* direction. The velocity in the y-direction obtained with the proposed model shows a good agreement with the laser doppler vibrometer measurements during the unloading phase (Fig 5B), although high frequency content is missing from the model (Fig 5C, D). The velocity response obtained later in time is less accurate. Overall the simple model captures the stages of motion measured by laser vibrometry well, which supports the conclusion that the snapping organ uses elastic recoil during unloading and resonates following the high-velocity motion.

**Reference**

1. Meirovitch L. Elements of vibration analysis*.* New York: McGraw-Hill; 1975.
